# Progress in the Development of Chitosan Based Insulin Delivery Systems: A Systematic Literature Review

**DOI:** 10.3390/polym12112499

**Published:** 2020-10-27

**Authors:** Francivandi Coelho Barbosa, Milena Costa da Silva, Henrique Nunes da Silva, Danyllo Albuquerque, Allyson Antônio Ribeiro Gomes, Suédina Maria de Lima Silva, Marcus Vinícius Lia Fook

**Affiliations:** 1 Department of Materials Engineering, Postgraduate Program in Materials Science and Engineering, Federal University of Campina Grande, Campina Grande PB 58429-900, Brazil; francivandi@gmail.com (F.C.B.); milecost@hotmail.com (M.C.d.S.); henrique.nunes.silva.eng@gmail.com (H.N.d.S.); danyllo@copin.ufcg.edu.br (A.A.R.G.); 2 Electrical Engineering and Informatics Centre, Postgraduate Program in Computer Science, Federal University of Campina Grande, Campina Grande PB 58429-900, Brazil; aaribgomes@hotmail.com; 3 Department of Materials Engineering, Federal University of Campina Grande, Campina Grande, PB 58429-900, Brazil

**Keywords:** delivery system, release system, insulin, chitosan, systematic review

## Abstract

Diabetes mellitus is a chronic disease that is considered a worldwide epidemic, and its control is a constant challenge for health systems. Since insulin had its first successful use, scientists have researched to improve the desired effects and reduce side-effects. Over the years, the challenge has been to increase adherence to treatment and improve the quality of life for diabetics by developing an insulin delivery system. This systematic review (SR) analyses experimental articles from 1998 to 2018 related to the development of the chitosan/insulin delivery system (CIDS). Automated support: Start tool was used to perform part of these activities. The search terms “insulin”, “delivery or release system”, and “chitosan” were used to retrieve articles in PubMed, Science Direct, Engineering Village, and HubMed. A total of 55 articles were selected. The overview, phase, model, way of administration, and the efficiency of CIDS were analyzed. According to SR results, most of the articles were published from 2010 onwards, representing 72.7% of the selected papers, and research groups from China publicized 23.6% of the selected articles. According to the SR, 51% of the studies were carried out in vivo and 45% in vitro. Most of the systems were nanoparticle based (54.8%), and oral administration was proposed by 60.0% of the selected articles. Only 36.4% performed loaded capacity and encapsulation efficiency assays, and 24 h (16.4%), 12 h (12.7%), and 6 h (11.0%) were the most frequent insulin release times. Chitosan’s intrinsic characteristics, which include biodegradability, biocompatibility, adhesiveness, the ability to open epithelial tight junctions to allow an increase in the paracellular transport of macromolecular drugs, such as insulin, and the fact that it does not result in allergic reactions in the human body after implantation, injection, topical application or ingestion, have contributed to the increase in research of CIDS over the years. However, the number of studies is still limited and the use of an alternative form of insulin administration is not yet possible. Thus, more studies in this area, aiming for the development of an insulin delivery system that can promote more adherence to the treatment and patient comfort, are required.

## 1. Introduction

Diabetes mellitus is a chronic disease that is considered a worldwide epidemic, and its control is a constant challenge for health systems worldwide. According to data publicized by the World Health Organization [1], the estimated number of people with the disease around the world has been increasing since 1980. The outlook is that this figure will have increased to 642 million by 2040, according to the International Diabetes Federation [2]. One of the treatments for this disease is the use of exogenous insulin, especially for patients with Type 1 diabetes mellitus, although insulin may also be required for patients with Type 2 diabetes mellitus [1].

Since insulin had its first successful use and started to be commercialized, scientists have constantly been developing new research to improve the desired effects and reduce the collateral ones. In the 1950s basal insulin began to be used, and in the 1990s more convenient and rapid-acting insulin was developed. The duration of basal insulin was improved with the development of glargine insulin, in 2000, and detemir insulin in 2004 [3]. Despite improvements in both basal and prandial insulin, the base treatments involve administration of daily subcutaneous injections, resulting in discomfort for the patient, and promoting non-adherence to the treatment [4]. For many years, thereby, science has been challenged to improve the quality of life and increase adherence to the treatment for diabetics, especially through the development of insulin delivery systems [5]. The aim of such release systems is to prolong and improve the drug delivery control by optimizing its therapeutic action with minimal side-effects [6]. One of the polymers which have been associated with insulin in an attempt to obtain an effective delivery system is chitosan. Chitosan matrix systems have been extensively explored, being one of the main research interests regarding the development of systems which enable the oral use of insulin [7,8,9,10,11,12,13,14]. However, oral insulin administration is limited by a number of factors, such as enzymatic degradation in the gastrointestinal tract and low permeability across the intestinal membranes [15]. Thus, other insulin delivery systems, such as the transbuccal [16,17], buccal [18], nasal [19,20,21], subcutaneous [22], and transdermic [23] have been studied.

Moreover, some systematic reviews related to insulin use and the effects of these treatments have been performed. Balena et al. [24] analyzed randomized controlled clinical trials of insulin/GLP-1 RA combined therapy, and examined the results of such combinations, as reported through observational and clinical practice studies. Another report compared the effects of oral hypoglycemic agents with insulin, in achieving glycemic control, and studied the maternal and perinatal outcomes in gestational diabetes [25]. Aiming to compare continuous subcutaneous insulin infusion and multiple daily insulin injections, Jeitler et al. [26] made a systematic review and meta-analysis comparing the effects of continuous subcutaneous insulin infusion, with multiple daily insulin injections, on glycemic control, risk of hypoglycemic episodes, insulin requirements, and adverse events, in type 1 and type 2 diabetes mellitus patients. Focusing on insulin treatment, Khunti and Millar-Jones [27] reviewed evidence of clinical inertia to insulin initiation and intensification, as well as the barriers, and potential solutions, in a free universal healthcare country.

Although the amount of research reviewing insulin treatment, and pointing out novel and varied chitosan/insulin delivery systems is increasing, to the best of our knowledge, there has been no study providing an overview of empirical evidence, as well as the main gaps and opportunities for future studies. In this sense, the main research objective herein was to provide a piece of integrated information from a set of primary chitosan/insulin delivery system related studies, which presented some empirical evidence on the field. Additionally, this study identifies some issues which require the support of new or further evidence, thus assisting in orientation of the scientific community for future research. Bearing this in mind, a systematic review (SR) was carried out, by collecting primary studies from 1998 to 2018 dealing with the development of chitosan matrix insulin release systems. This SR was performed following the guidelines provided by Kitchenham [28], and provides a state-of-the-art overview of chitosan matrix insulin release systems. This scientific method is a secondary study that identifies, evaluates, and interprets all the available studies on a particular research question, or topic area, or phenomenon of interest. It promotes a summary of the existing evidence and provides a background for new studies using a predefined search strategy [29]. In this perspective, the main contributions of our study are: (I) the systematic identification and analysis of the existing research, pointing out the methods, techniques, and methodologies used, final product forms and release times obtained from the system, and (II) the provision of knowledge for deriving new test hypotheses and identifying issues for future research.

## 2. Materials and Methods

### 2.1. Search Strategy

**Figure 1:**
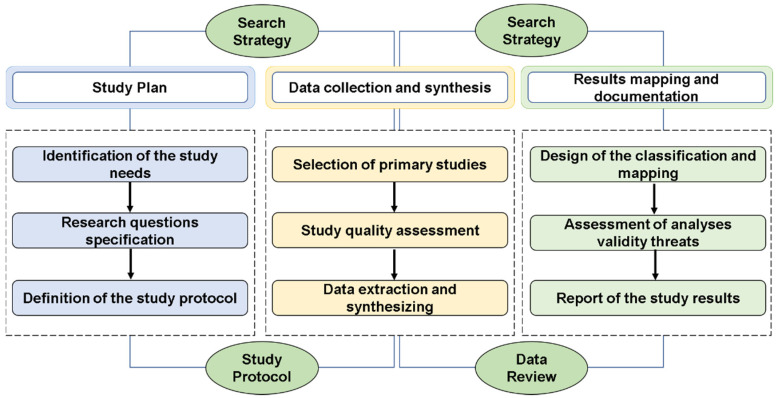
Steps followed in the systematic review (SR) research methodology.

The systematic review (SR) was delimited by following, with some modifications, the guidelines by Kitchenham [28]. This empirical strategy has the advantage of minimalizing the bias of the selection, identification, and systemization of the data and results. The steps followed in the study were: (I) planning; (II) data collection and synthesis; and (III) result mapping and documentation (Figure 1). In the planning stage a pilot study was executed to define the study parameters (needs, research questions, and protocol) and verify the feasibility of the research. For the data collection and synthesis, the activities of selection, evaluation of the studies, and data extraction were performed. An automated support, Start tool (Supplementary material), was used to perform part of these activities. In the mapping and result documenting stage the classifying and mapping of the selected studies was performed, and the main results were presented.

### 2.2. Research Questions

In this SR, intending to extract as much data as possible, the research questions (RQs) were defined so as to investigate and outline key aspects of the research (Table 1). Proposed solutions to functional chitosan/insulin delivery systems (CIDS) were discussed in the selected articles. The main objective of the questions was to identify problems and innovative solutions in the development of the delivery systems, aiming to inform future research.

Using the RQ the main points of the selected articles were extracted: RQ1, temporal progress for the development of the CIDS; RQ2, classification and identification of the models developed; RQ3, alternative administration forms proposed; RQ4, efficiency of the methods used to prepare the delivery system; and, RQ5, the period of insulin release.

### 2.3. Primary Study Selection

To define the search terms, the RQs were used in order to: (i) detect keywords used in the articles to generate the search strings (string composition), (ii) find and identify studies with chitosan matrix-based insulin delivery systems as the primary study focus, (iii) do a secondary study on the subject from 1998 to 2018. The relevant terms were then extracted from paper titles, abstracts, and keywords. The search string used was as follows: ((chitosan) and (insulin) and (delivery system)) and ((controlled delivery system) or (drug delivery) or (release system)). The foregoing search string was customized for each database to obtain effective results.

### 2.4. Supplementary Material

To guarantee the authenticity and reproducibility of this study, additional material was created. It contains information on 55 experimental articles from 1998 to 2018 which deal with the development of chitosan/insulin delivery systems (Table S1), strings, and the Start tool used in the development and data analyses.

## 3. Results and Discussion

### 3.1. Overview of the Articles

**Figure 2:**
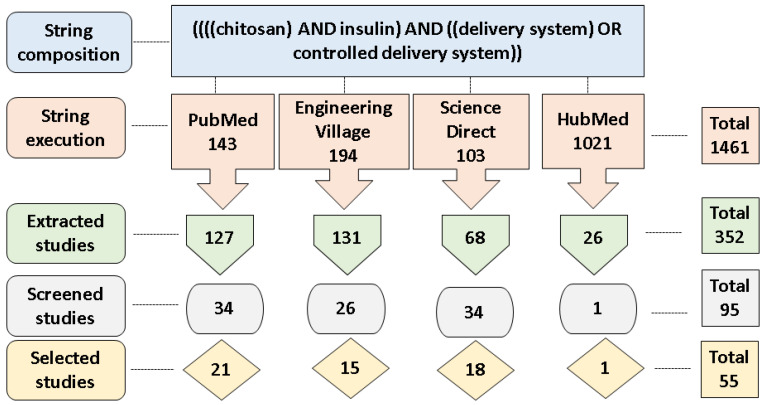
Scheme of the literature search process to obtain the articles used in the SR.

In this section, an overview of the selected articles is presented. A comprehensive data set is made available in the supplementary material. To date, 1461 studies have been found in the primary study search process (string execution). The cut-off point (the pre-defined keywords found in the title, abstract, and keywords of the articles) was applied to the Start tool, and 352 studies were obtained. For the pre-selected papers, the title keywords and abstract were analyzed, yielding 95 studies. For these, reading of the introduction, methodology, and conclusion was undertaken, and, after this final selection step, 55 studies were obtained. A summary of these steps is depicted in Figure 2. The complete data set is available in the supplementary material.

**Figure 3:**
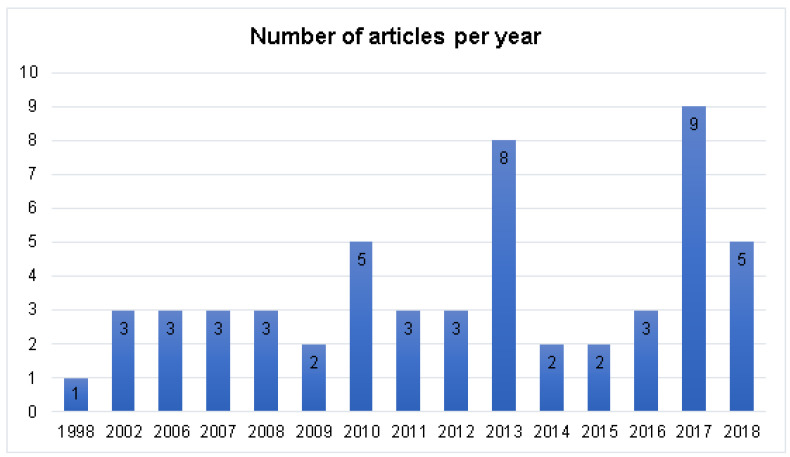
General view of the number of publications per year.

Considering the year of publication (Figure 3), only one of the studies was performed in 1998, and three in 2002. After that, the publications were only resumed in 2006, with most articles being published from 2010 onwards (72.7% of the selected papers). There was a concentration of publications in the years 2010 (5), 2013 (8), 2017 (9), and 2018 (5). The results shown in Figure 3 point out that, although the development of new routes for insulin administration is not a recent idea, the advance and improvement of chitosan/insulin release systems has been occurring more intensively since 2010. However, the number of publications is still limited, so more studies in this research line should be conducted.

**Figure 4:**
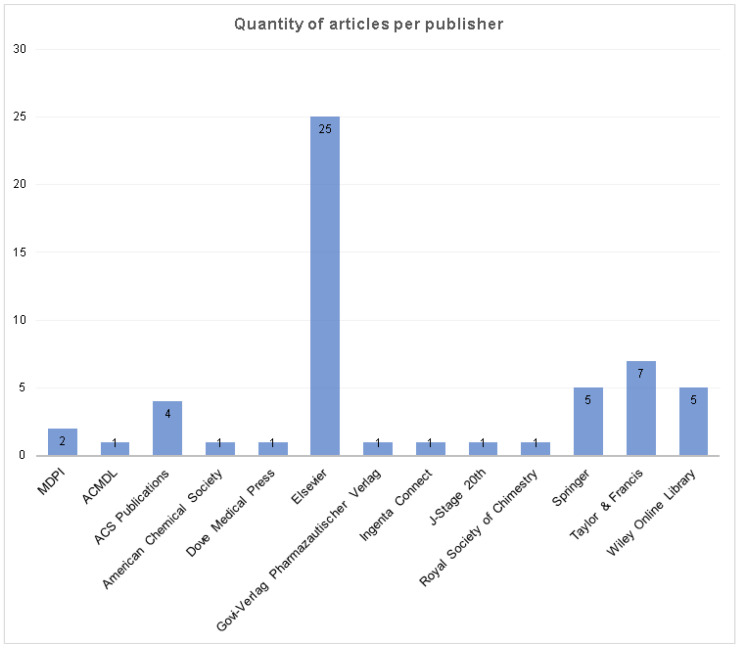
Amount of articles per publisher, considering the sample.

It was possible to identify 13 publishers’ online databases with articles on the subject “chitosan/insulin delivery system” (Figure 4). Elsevier had the greatest amount of publications (45.4%), followed by Taylor & Francis (12.7%), Springer (9.1%), Wiley Online Library (9.1%), ACS Publications (7.3%), and MDPI (3.6%). The other seven online databases had just one publication on the subject (supplementary material).

**Figure 5:**
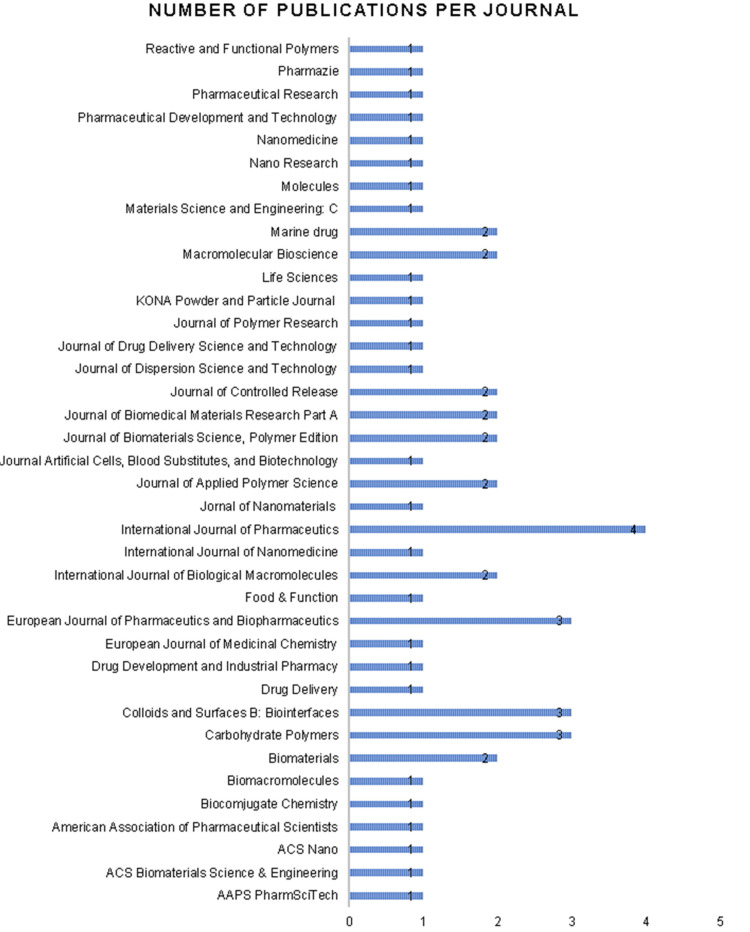
Number of publications per Journal.

Considering the source in which the selected articles were published, 38 different journals were found (Figure 5). Most of the journals were focused on the pharmaceutical (31.6%), and material science and/or polymer (31.6%) areas, although articles about chitosan/insulin release systems had also been published in journals comprising several fields, such as chemistry, biotechnology/biology, and medicine, among others (supplementary material). The journals with the largest number of publications were the International Journal of Pharmaceutics (10.5%), the European Journal of Pharmaceutics and Biopharmaceutics (7.9%), the Colloids and Surfaces: Biointerfaces (7.9%), and the Carbohydrate Polymers (7.9%).

Combining the results obtained from the publishers (Figure 4) and journals (Figure 5) it can be inferred that the subject has importance and breadth for many research areas, and that the interest and involvement in the development of chitosan/insulin release systems for new routes of insulin administration is a challenge yet to be overcome.

**Figure 6:**
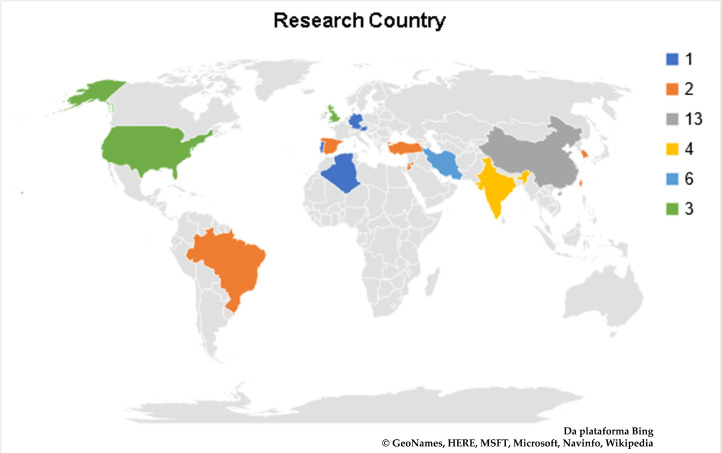
Overview of the research countries from the selected articles.

The selected articles were produced by many research groups from several countries (Figure 6). Most articles were produced by Chinese research groups, with 13 publications (23.6%); followed by Iran (11.0%) with 6 publications; Jordan (9.1%), 2 publications by their own research groups, and 3 in association with other countries; and India (9.1%), with 4 publications from national groups, and 1 associated with Portugal. Other articles were produced in 19 different countries, individually or in association, and can be consulted in the supplementary material.

**Figure 7:**
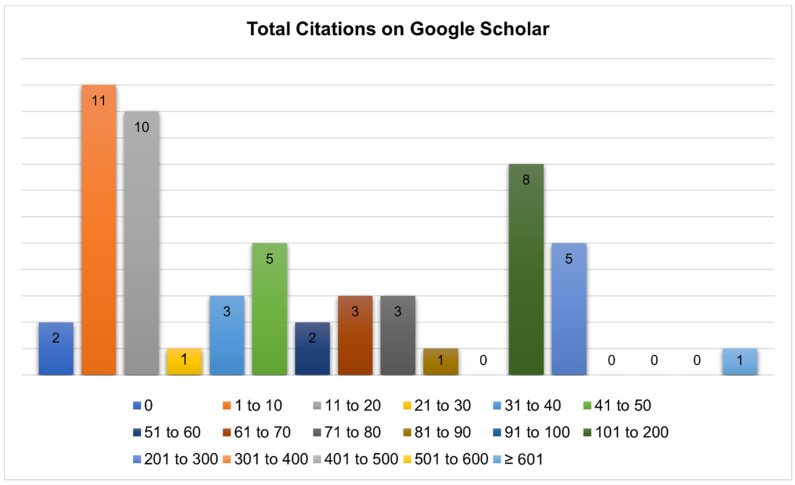
Quantity of papers versus number of citations on Google Scholar.

The categorization of the articles citations was performed using an online database which recovered all the selected articles (Google Scholar), and is presented in Figure 7. Only two articles did not have citations. While 38.2% of the publications had from 1 to 20 citations, and 32.7% had between 21 and 90 citations. It is worth pointing out that, 23.6% of the selected articles had 101 to 300 citations, and just one paper had more than 600 citations.

To verify the academic impact of the selected articles a categorization was made using Google Scholar citations (Figure 7). The papers not bearing citations were from 2018. This is probably due to their recent publication dates. The publication cited by more than 600 articles was developed in China in the year of 2002, and was published in the International Journal of Pharmaceutics [10]. Considering the results, this study has great importance since most of the selected articles have more than 20 citations on Google Scholar. Further information can be consulted in the supplementary material.

### 3.2. Research Questions (RQ)

The main points presented herein are: temporal progress for the development of the chitosan/insulin release systems (RQ 1); classification and identification of the models developed (RQ 2); alternative administration methods proposed (RQ 3); efficiency of the methods used to prepare the delivery system (RQ 4); and the insulin release period (RQ 5).

#### 3.2.1. RQ1. At What Stage of Development Is the Chitosan/Insulin Delivery System?

**Figure 8:**
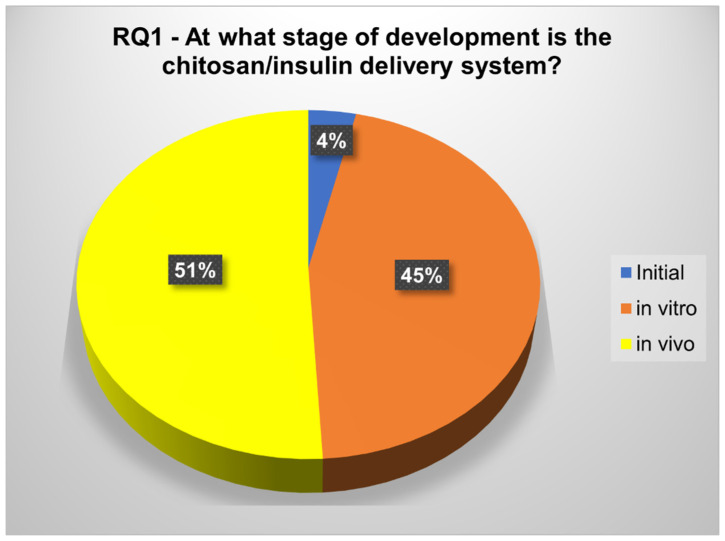
Development stage of the studies according to RQ1.

The development progress of the chitosan/insulin release systems was classified into in vivo, in vitro and initial (Figure 8). From the selected articles, only 4% (2) were classified in the initial phase, one being from 1998 and the other from 2013, 45% described in vitro insulin release studies, and most (51%) reported in vivo experiments.

At this point, the chitosan/insulin release systems’ development progress was investigated. The phases were classified into in vivo (papers describing experiments with animals); in vitro (papers reporting only in vitro release studies); and initial (papers presenting neither in vivo nor in vitro description) (Figure 8). The results account for a high number of papers at an advanced phase. Although 39.3% of these articles do not present previous release studies, they describe experiments demonstrating the glucose control level using the developed system (supplementary material). The evaluation of the drug release system is important to obtain the characteristic of the developed system, and this information can direct the method and timing of the administration for an in vivo experiment. Therefore, a drug release system aims to prolong and improve the drug administration control, by optimizing its therapeutic action, with minimal side effects [6].

The articles classified as initial aimed at developing new methods of creating a CIDS. Bernkop-Schnürch et al. [30] and Robles et al. [31] investigated the interaction of insulin with unmodified chitosan and hydrophobized chitosan in polyelectrolyte complexes. Both studies observed that the chitosan/insulin polyelectrolyte complex showed high stability, high interaction between insulin and chitosan, and greater constant diffusion for low and moderate chitosan/insulin proportions, when compared to the hydrophobic ones.

Half of the works classified in the in vitro stage were developing chitosan nanoparticles for oral insulin administration [8,12,13,16,32,33,34,35,36,37,38,39,40]. The authors were aiming at drug delivery systems resistant to the gastric environment, and able to protect insulin from enzymatic activity. Seeking to verify this property, some of them performed in vitro tests on simulated gastric fluid and/or simulated intestinal fluid. Zhang et al. [35] obtained an alginate/chitosan nanoparticle system for insulin protection by complexation with cationic β-cyclodextrin polymers. The nanoparticles were effective in protecting insulin under simulated gastrointestinal conditions. In simulated intestinal fluid, insulin was released over 6 h. Sahoo et al. [39] developed chitosan and carboxymethylated iota-carrageenan insulin-loaded nanoparticles. Insulin release was low (4.91 ± 0.24%) in simulated gastric fluid and high (86.64 ± 2.2%) in simulated intestinal fluid during a 12-h release study; demonstrating release properties responsive to the environmental pH. Lancina et al. [16] obtained stable chitosan-pectin nanoparticles and microparticles in simulated gastric fluid (pH 1.2). Then, the particles successfully released insulin into a simulated intestinal fluid (pH 6.8) over 2 h.

The in vivo tests were used to confirm if the developed delivery systems could maintain insulin pharmacological activity in living organism conditions. Streptozotocin-induced diabetic mice are a conventionally used model to test insulin delivery systems [41,42,43,44,45,46,47]. Al-Remawi et al. [48] and Di et al. [47] used this model in studies to verify the oral absorption and ultrasound-triggered insulin release, respectively. Al-Remawi et al. [48] developed insulin-chitosan polyelectrolyte complexes associated to lecithin liposomes. In this study a significant reduction in blood glucose levels was observed 2 h after oral administration, and the effect was prolonged for 8 h, indicating the ability of the liposomal vesicles to enhance the pharmacological activity of insulin. Di et al. [47] evaluated the performance of poly(lactic-co-glycolic acid) (PLGA) nanoparticles loaded in chitosan microgel as an injectable ultrasound-triggered insulin release system. The microgel was subcutaneously injected into the dorsum of each mouse, and the focused ultrasound was performed. Ten minutes after the ultrasound treatment, a reduction in blood glucose level was detected, and the result obtained was consistent the following week.

#### 3.2.2. RQ2. What Are the Release Systems Developed?

The identification and categorization of the proposed models were described and accounted for, even if they were developed by only one research group.

**Figure 9:**
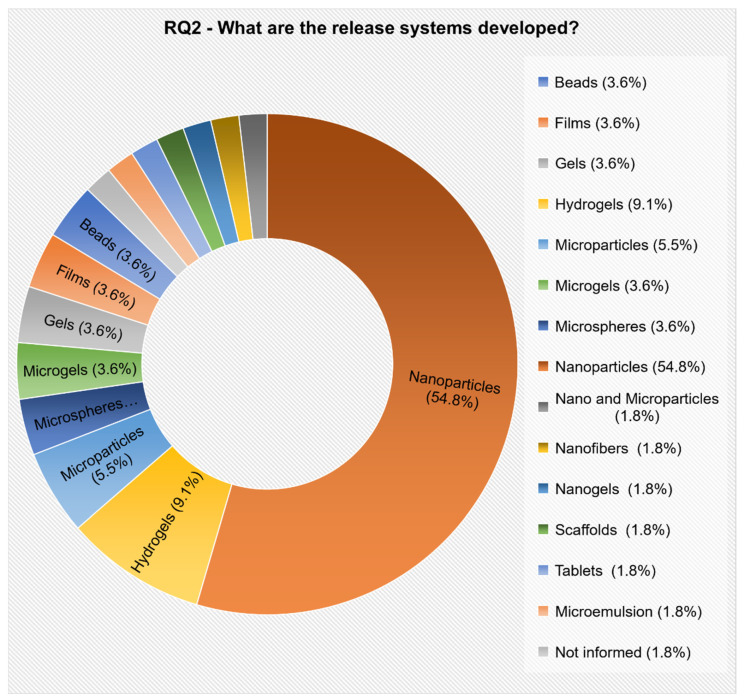
Release system models presented in the selected articles, according to RQ2.

Fourteen different models were identified (Figure 9). The prevalent model, developed by most researchers, was nanoparticles (54.8%), followed by hydrogels (9.1%), microparticles (5.5%), microspheres (3.6%), microgels (3.6%), gels (3.6%), films (3.6%), and beads (3.6%). The other models proposed were cited by only one paper each (supplementary material).

At this point, the proposed models presented in the selected articles were identified and categorized (Figure 9). Observing the nanoparticle models it can be noticed that 76.7% of the release systems were developed for oral use. This is probably due to the limitations of the oral delivery of protein drugs, such as enzymatic degradation in the gastrointestinal tract, and low permeability across the intestinal membranes, which results in low oral bioavailability. The polymeric nanoparticles can contribute to enhancing intestinal absorption of insulin by enabling an intimate contact of the drug with the intestinal mucus layers [49].

##### Nanoparticles

Nanoparticles were the most reported insulin release system (54.8%). A study about insulin stability in chitosan polyelectrolyte complex was developed by Al-Kurdi et al. [37]. Nanoparticles with varying degrees of low chitosan molecular weight were evaluated to improve insulin stability. Insulin was stable in a polyelectrolyte complex consisting of insulin and low molecular weight chitosan, in the presence of a tris-buffer at pH 6.5. The stability of insulin increased with the decreasing molecular weight of chitosan. Furthermore, 13 kDa was the most efficient chitosan molecular weight for enhancing the physical and chemical stability of insulin.

Avadi et al. [34] proposed a new insulin delivery nanoparticulate system, based on ionic gelation between chitosan and Arabic gum. The main objective in the use of Arabic gum was to protect the insulin from the harsh gastric and enzymatic intestinal environments. Various formulations were prepared using 2^3^ factorial designs, varying the chitosan, Arabic gum, and insulin concentrations. The association efficiency was the parameter to be optimized with the factorial design. The authors observed that the efficiency of the association increases with the increasing concentration of Arabic gum.

Krauland et al. [50] developed a new type of nanoparticle based on chitosan and carboxymethyl-cyclodextrin for insulin release. The nanoparticles were prepared with mixtures of chitosan and carboxymethyl-β-cyclodextrin via the ionotropic gelation technique in the presence of tripolyphosphate. These nanoparticles were stable in simulated intestinal fluid (pH 6.8 at 37 °C) for at least 4 h. However, the authors concluded that the developed systems may be candidates for nasal and oral insulin release.

##### Gel (Hydrogel and Microgel)

A chitosan/polyvinyl alcohol hydrogel for nasal insulin delivery was developed by Agrawal et al. [20]. The hydrogels were prepared by mixing the chitosan and PVA solutions in different proportions, and the final pH of the solutions was adjusted with 1.0 M NaHCO_3_ to a pH close to neutral. The insulin was added in the formulated delivery system so that the final solution contained 1 IU insulin per 200 µL of the solution. The prepared hydrogel was liquid at room temperature, and after being incubated at 37 °C for 12 min it showed a gelation transition to non-flowing hydrogel. The in vitro release of insulin test showed a maintenance of glucose levels for 6 h. In the in vivo study it was observed that the maximum reduction was at four hours after administration. This means that a slow-release was obtained through the gel network.

Gu et al. [44] developed monodisperse microgels consisting of chitosan matrix, enzyme nanocapsules, and recombinant human insulin. Glucose-specific enzymes were covalently encapsulated into the nanocapsules to improve enzymatic stability by protection from denaturation and immunogenicity. The authors reported that enzymatic conversion of glucose into gluconic acid in the noncovalent cross-linked polymeric matrix reduced the microenvironmental pH, resulting in swelling and dissociation of the microgels, and increasing the release rate of insulin.

A biodegradable, biosensitive in situ gelling system based on chitosan was developed by Kashyap et al. [41]. The authors’ objective was to devise a pulsatile insulin delivery technique in response to the glucose concentration, and as such performed in vitro and in vivo studies. The gel was prepared using chitosan and phosphate disodium, with addition of glucose oxidase, peroxidase, and insulin. They reported that pulsatile insulin release was glucose dependent, when conducted in 3 mg/mL glucose medium, and that this condition was maintained for 28 h, although not much release was seen after 4 h. Degradation was faster in insulin-loaded gels, and could not be felt 3–4 days post-injection. In vivo assay showed, when 3 IU/Kg was administrated, plasma glucose levels (PGL) was decreased to as low as 1.12 (mg/mL) by 4 h, and it returned to basal levels by 16 h.

##### Microparticles (Microspheres and Beads)

Water-soluble chitosan/insulin/tripolyphosphate microparticles, with higher association efficiency at pH 4.0 (48.28 ± 0.90%), were prepared by Wu et al. [51]. They produced the chitosan/insulin/tripolyphosphate microparticles using the polyelectrolyte complexation method. The authors investigated in vitro insulin release in simulated gastric and intestinal media, which presented three phases: (1) an initial burst release of 50% (in 2 h), (2) a plateau for the following 12 h, and (3) a constant sustained release of the remaining drug.

Jose et al. [52] developed chitosan microspheres loaded with insulin, produced through the emulsion cross-linking method, with glutaraldehyde as cross-linker. Insulin-loaded chitosan microspheres showed swelling ratios between 1.30 and 2.30. In vitro insulin release profiles showed a burst release pattern in the first 3 h and then a controlled release in the following 5–6 h. Chitosan microsphere formulations gave a good fit to the Higuchi model, with R2 ≈ 0.98, thus the drug release mechanism was assumed to be diffusion controlled.

Insulin-loaded alginate/chitosan blend gel beads were prepared by Tahtat et al. [53]. The homogeneous solution of alginate/chitosan was dual crosslinked with CaCl_2_ and glutaraldehyde solutions to improve mechanical properties, so as to withstand the simulated gastric and intestinal fluids. They reported that the presence of chitosan in the blend beads decreased the cumulative insulin release in gastric media, and enhanced the behavior of alginate/chitosan beads in intestinal medium due to the crosslinking. The cumulative insulin release from alginate/chitosan beads reached 90.5% within 6 h.

##### Films

Studies which developed chitosan films as a controlled-release insulin system proposed buccal or transbuccal administration.

Giovano et al. [18] aimed to develop chitosan-based mucoadhesive films loaded with PEG-b-PLA/insulin nanoparticles for buccal insulin administration. Free insulin dispersed within chitosan films was completely released (98 ± 14%) after 6 h of incubation. Insulin was released over a more prolonged period when it was first encapsulated into nanoparticles before incorporation into the films, with an initial phase release of 51 ± 14% of total peptide in 72 h. A modulated release was observed, with 70% of encapsulated insulin released after 360 h. The films showed mucoadhesion properties.

Cui et al. [17] developed a bilaminated film, composed by a mucoadhesive layer (chitosan-ethylenediaminetetraacetic acid hydrogel film) containing insulin, and an impermeable protective layer made of ethylcellose. The films showed a mucoadhesive force of the hydrogel which remained over 17,000 N/m^2^ for 4 h in the in vitro test, using a simulated oral cavity. A hypoglycemic effect was reported, following buccal administration to healthy rats, achieving a 17% pharmacological availability compared to subcutaneous insulin injections.

#### 3.2.3. RQ3. What Are the Main Alternative Ways of Administration Suggested?

The alternative ways of administration proposed by the selected papers were categorized.

**Figure 10:**
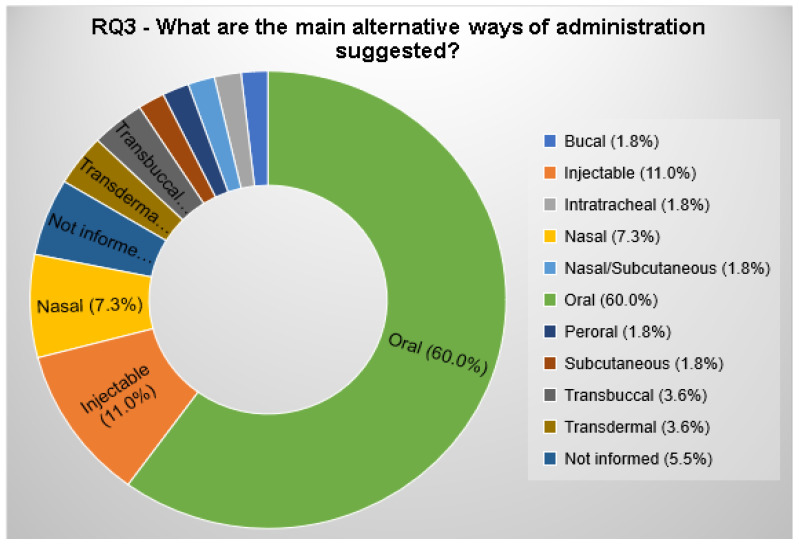
Alternative ways of administration suggested by the selected articles.

Conducting this systematic review (SR) it was possible to verify that 60.0% of the selected articles aimed to produce release systems for oral administration (Figure 10). The second most proposed way of administration was the injectable one (11.0%); followed by nasal (7.3%), Transdermal (3.6%), and transbuccal (3.6%) administration. A total of 5.5% of the selected articles do not include the alternative method of administration. The other alternative ways of administration can be seen in Figure 10 and in the supplementary material.

In this topic, all the proposed ways of administration were identified and categorized, including injectable administration.

Despite being a challenge, because of gastrointestinal limitations, like harsh acidic stomach, extensive enzymatic degradation, mucosal surface of the intestine, and tight junction in between the epithelial cells [34], oral administration was the most proposed way to administrate chitosan/insulin in the delivery systems (CIDS) (60.0%).

The oral approach is seen as the most attractive due to convenience and high patient compliance. Another potential advantage of oral administration is to simulate the natural hepatic activation of insulin instead of the systemic insulin ratio involved in subcutaneous injections [33]. Furthermore, oral administration would provide a lower cost to industry and patients [34].

Curiously, the second most proposed way of administration was the injectable (11.0%); whereas, one of the most current justifications for the development of a chitosan/insulin release system is as an alternative way of administration because, according to the American Diabetes Association [4], conventional insulin treatment involves subcutaneous injection, which can cause discomfort to the patient, and may promote non-adherence to treatment.

Relating the ways of administration to the developed models, the gels (gel, hydrogel, and microgel) were the most developed system for the injectable forms (83.3%); perhaps because the injectable way affords more stability and facility to administrate the system. Di et al. [47] prepared a chitosan microgel that encapsulated insulin loaded PGLA nanoparticles. The microgel can temporarily store insulin released from the nano capsules, and can release insulin for 240 h. Other researchers incorporated Insulin and a pH sensitive nanoparticle, composed of glucose oxygenase and catalase, into a chitosan microgel. In this work, the incorporation of the enzymes into microgels facilitated the insulin release, and the blood glucose level control was retained for 12 h [44].

Khodaverdi et al. [54] and Ghasemi Tahrir et al. [45] formulated a hydrogel of chitosan and glycerol phosphate for insulin release, both detected the gelation dependences on the concentration of insulin and glycerol phosphate, and the stability of insulin after being released from the system. Ghasemi Tahrir et al. [45], in an in vivo assay, observed significantly low blood glucose levels for 120-h post administration for a system with 8% of Glycerol phosphate, although normal levels of blood glucose were not achieved.

To prepare a CIDS, to a gel of chitosan and glycerol phosphate glucose oxidase, peroxidase and insulin were added. After injection a glucose dependent pulsatile insulin release was observed, and this condition was maintained for 28 h. Although in the in vivo assay the glucose levels were reduced for 4 h, they returned to basal levels for 16 h [41].

The proposal of a nasal method of administration was considered due to the advantages offered, such as large surface area available for drug absorption, high vascularization, low dose requirement, and quick onset of the pharmacological activity [20,21]. However, macromolecular drugs, like insulin, show low bioavailability when administrated via nasal methods. The disadvantages of this administration are high enzymatic activity, limited permeability of the mucous barriers, and mucociliary clearance [21,50]. Of the researchers who chose nasal administration for the CIDS, 75% developed nanoparticle systems [21,50,55]. This option can be explained by the capacity of nanoparticles to cross and transport associated molecules through the mucous barrier, and maintain the stability of the drug.

Zhang et al. [21] developed PEG-g-chitosan nanoparticles incorporated with insulin and, in the in vivo assay, conducted with rabbits through nasal administration, the nanoparticles showed a reduction in blood glucose of 68% in four hours after administration in relation to base line. The researchers suggested that the nanoparticles improved the insulin absorption through the nasal mucosa. Other authors proposed the use of chitosan and chitosan/alginate nanoparticles loaded with insulin dissolved in acetate buffer. The nanoparticles were administrated intranasally in rabbits; the system allowed the nasal absorption of insulin, and results demonstrated the capacity of the nanoparticles to reduce glucose levels [55]. Krauland et al. [50] loaded insulin in chitosan/carboxymethyl-*β*-cyclodextrin nanoparticles. The in vitro assay demonstrated that the nanoparticle are stable for 2 h and released 84%–97% of the loaded insulin in the first 15 min. Despite the low stability and fast release, the authors concluded that the system was a possible candidate for improving the absorption of macromolecules in nasal administration.

Transdermal, and transbuccal administration were each proposed by 3.6% of the selected articles (Figure 10). The transdermal system confronts the limitation of the low permeability into the stratum corneum of the skin. Even if this limitation is overcome, the transdermal administration can maintain insulin stability and avoid the first-pass effect [23]. Similarly, the buccal mucosa does not allow a fast protein drug diffusion, being even more permeable than skin. Nevertheless, with the use of some strategies, the transbuccal administration was able to avoid a rapid systemic circulation drug reach, as well as the first-pass effect [16].

To develop a transdermal administration Zu et al. [23] prepared a chitosan–polyvinyl alcohol hydrogel blend loaded with insulin. The in vitro assays demonstrated that all the components were compatible, and the system obeyed Fick’s first law, and had a high permeation rate. Lee and Kang [56] developed biodegradable nanoparticles to carry insulin via transdermal administration. The authors obtained a better reduction in glucose levels when dimethylsulfoxide (DMSO) was used, although the normal level was not achieved.

A porous, flexible bilaminated film, with a layer of Hydrophobic ethylcellulose, and a mucoadhesive layer of chitosan-ethylenediaminetetraacetic acid (EDTA) was developed aiming at transbuccal administration of insulin. The film had lower mucoadhesive force, the accumulative release amount of insulin was greater than 70% after 5 h, and the in vivo assay showed a sustained reduction of blood glucose levels over 5 h [17]. Objectifying a transbuccal administration, Lancina et al. [16] produced chitosan nanofibers through electrospinning, using poly (ethylene oxide) as a carrier molecule. The fibers presented significantly faster insulin release kinetics, and the model had Fickian diffusion. After the electrospinning the insulin stability presented a reduction and, in an ex vivo assay, the fiber delivered around one third of the total insulin into the acceptor chamber after 6 h.

#### 3.2.4. RQ4. What Is the Total Amount of Insulin Loaded and the Encapsulation Efficiency of the Release System?

**Figure 11:**
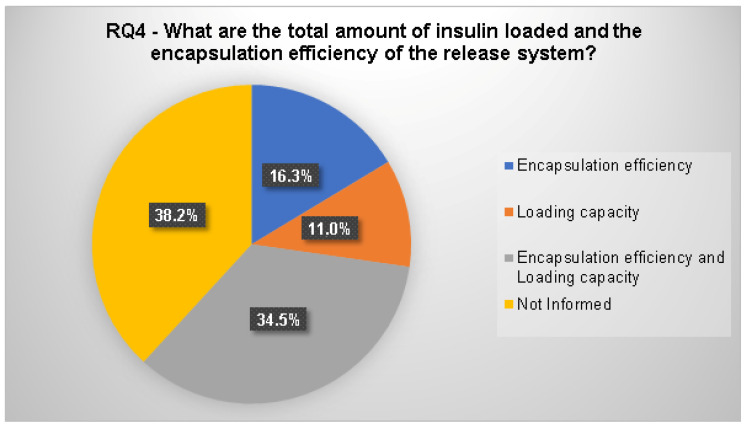
Classification of the articles according to the presence or absence of the release system load capacity and encapsulation efficiency analyses.

The methods used to prepare the delivery systems were classified by the presence or absence of the load capacity and encapsulation efficiency assays (Figure 11). The total amount of insulin loaded, and encapsulation efficiency can be consulted in the supplementary material.

In this study, 34.5% of the papers executed load capacity and encapsulation efficiency assays (Figure 11). The quantity of articles that did not inform the load capacity and encapsulation efficiency amounted to 38.2%. From the selected articles, 27.2% performed only load capacity or encapsulation efficiency (10.9% and 16.3%, respectively) assays.

This section presents the analyses of the methods used to prepare the delivery systems by classifying the selected studies according to the presence or absence of the load capacity and encapsulation efficiency assays.

The encapsulation efficiency assay is an important feature for release systems because the amount of encapsulated drug establishes the performance of the delivery system by influencing the rate and extent of drug release. The encapsulation efficiency depends on the load capacity because it determines the amount of drug incorporated into the release system [57]. Even so, the amount of articles that did not inform about the load capacity and encapsulation efficiency (38.2%) was greater than those in which both assays were performed (34.5%). From the selected articles in which load capacity and encapsulation efficiency assays were executed, 36.8% also developed in vivo, and 63.2% in vitro insulin release assays.

The load capacity and encapsulation efficiency were analyzed in different CIDS. Nano particles were developed and analyzed by many authors [8,12,21,32,34,37,38,48,50,58,59,60]. Avadi et al. [34] developed insulin nanoparticles, using chitosan and Arabic gum through the ionic gelation method, to evaluate the influence of the components in the encapsulation efficiency. The results demonstrated an increase in the encapsulation efficiency with the increase of Arabic gum concentration, although this effect was less important than the effect of the chitosan concentration. The nanoparticle load capacity was between 25% and 35%, and the insulin was in vitro, released for 4 h.

#### 3.2.5. RQ5. For How Long Did the System Release the Insulin?

**Figure 12:**
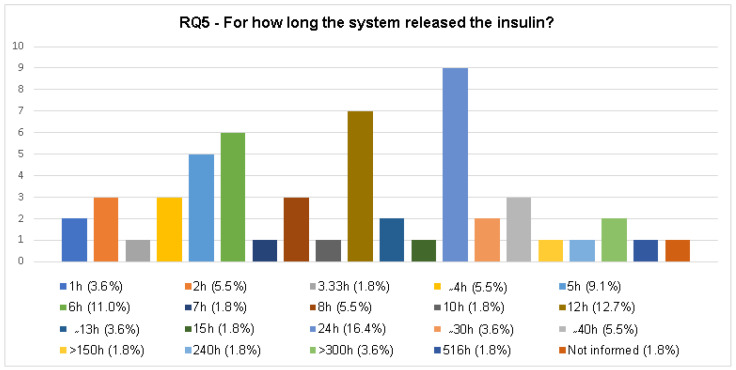
Categorization of the insulin release time from the chitosan/insulin release systems developed in the selected articles.

The release of insulin from the chitosan/insulin systems is presented in Figure 12. All the information about the insulin release was considered, whether the experiment was performed in vitro or in vivo. The full categorization is presented in Figure 12 and more information is available in the supplementary material.

In this section the insulin release of the chitosan/insulin deliver systems was identified and categorized. Analyzing insulin release time, there were nineteen different ones (Figure 12). In the majority of the articles (98.2%) the insulin release assay was performed. The articles which did not inform the release time were classified in the initial phase, and they did not perform the load capacity and encapsulation efficiency analyses either.

It is important to highlight that the articles’ reported insulin release time was higher than 100 h (Figure 12). A research group from Iran developed a chitosan/insulin release system that releases insulin for more than 150 h. This assay was conducted in vivo with a hydrogel system, and using the injectable method of administration [45]. An article from the United States reported a microgel chitosan/insulin release system tested in vitro an in vivo, which released insulin for 240 h, and the system used injectable administration [47]. Another study from an Iranian research group developed a hydrogel chitosan/insulin release system that released insulin for more than 300 h. The experiment was made in vitro, and the alternative way of administration proposed was the injectable way [54]. Although the developed systems have shown long periods of insulin release, the way of administration is the same as conventional administration, injectable, and can cause identical problems.

Aiming at a different way of administration, a group from the United Kingdom proposed a chitosan/insulin release system as a buccal film that releases insulin for more than 300 h, according to their in vivo test [18]. Other investigations from an Indian research group created a nanoparticle chitosan/insulin release system for oral use and, according to in vitro and in vivo assays, the insulin was released for 516 h [61]. However, the load capacity of the system was not revealed. More information is available in the supplementary material.

## 4. Risk of Biases

In this study, the strategies of Albuquerque et al. [62] and Kitchenhan et al. [29] were used to conduct the SR. As any empiric study, the SR had limits in approach, which must be considered for analyzing the potential impact of the validity threats. Three validity threats, associated with distinct activities of this SR, are presented herein.

### 4.1. Primary Study Identification

Biases were minimized in the search strategy by recuperating as many relevant studies as possible. Another strategy used was the search of literature based on relevant terms and their combination in the search string (supplementary material). These search strategies allow for the finding of a comprehensive series of relevant studies.

### 4.2. Quality of Studies and Data Extraction Consistency

The quality of the SR is directly related to the quality of the recovered studies. So, it is important to minimize the quality menace for the selected studies, and to guarantee a consistent representation of data extracted from these studies. For this study the bias was settled. As shown in Figure 4, most of the selected articles have more than 20 citations on Google Scholar.

### 4.3. Data Synthesis and Results Reporting

A limited number of searches is a limitation for data synthesis and results reporting. However, there are many search groups in many countries working with chitosan/insulin release (Figure 6), which is the main theme of this SR. Therefore, the present SR has high validity, considering the use of a systematic and recommended technique, and a pilot study to refine the scope of the review.

## 5. Conclusions

In this work, a review was carried out using a systematic review (SR) of experimental articles from 1998 to 2018 which deal with the development of chitosan/insulin delivery systems. The results were presented in illustrated figures and tables to provide a full comprehension of the SR process, and with the objective to systematize and promulgate knowledge about the development of chitosan/insulin release systems. This SR complements the existing research, since the systematic study provides a new exploratory framework, arguments, and enhanced theories and conclusions. In total, 55 experimental articles related to chitosan/insulin release systems from 1998 to 2018 were selected. Most articles were published from 2010 onwards, representing 72.7% of the selected papers, and research groups from China publicized 23.6% of the selected articles. Most articles have more than 20 citations on Google Scholar (60.0%). The study also shows the advanced development stage of the chitosan/insulin release systems, with 51% of the studies being in the in vivo and 45% in the in vitro phases. The majority of the investigated systems were nanoparticles (54.8%), and oral administration was proposed by 60.0% of the selected articles. Only 36.4% performed loaded capacity and encapsulation efficiency assays, and 24 h (16.4%), 12 h (12.7%), 6 h (11.0%), 5 h (9.1%), 2 h (5.5%), 4 h (5.5%), 8 h (5.5%), and ∼40 h (5.5%) were the most frequent insulin release times. In conclusion, an increase in studies related to new methodologies of chitosan/insulin release systems, for the encapsulation and release of insulin over the years, was laid bare, with the aim of promoting improvement in the patients’ quality of life. However, the number of studies is still limited, and more studies in this research area are necessary. The use of alternative methods for insulin administration is not yet possible, however, the development of new ones can save many lives, since it can promote more adherence to the treatment, and supply further comfort to the patient.

## Figures and Tables

**Table 1:** Research Questions.

**RQ**	**Description**	**Motivation**
RQ1	At what phase is the development of the chitosan/insulin delivery system?	This question provides a starting point to investigate the temporal progress of the development of chitosan/insulin release systems.
RQ2	What are the release systems developed?	The aim was to classify and identify the models developed from 1998 to 2018.
RQ3	What are the main alternative ways of administration suggested?	This question aims to identify the alternative administration forms proposed.
RQ4	What is the total amount of insulin loaded and the encapsulation efficiency of the release system?	This question intends to investigate the efficiency of the methods used to prepare the delivery system.
RQ5	For how long did the system release insulin?	The answer to this question allows knowledge of how long the systems can control glucose levels.

## Supplementary Materials

The following are available online at https://www.mdpi.com/2073-4360/12/11/2499/s1. Table S1: Information on 55 experimental articles from 1998 to 2018 which deal with the development of chitosan/insulin delivery systems.
