# Supplementary material for: Progress in the Development of Chitosan Based Insulin Delivery Systems: A Systematic Literature Review
Source: Polymers (Basel). 2020 Oct 27;12(11):2499. doi: 10.3390/polym12112499 (PMC7692135; doi:10.3390/polym12112499)
Supplement: Supplementary file 1 [file polymers-12-02499-s001.pdf]

Supplementary Material

Table S1: Information on 55 experimental articles from 1998 to 2018 which deal with the development of chitosan/insulin delivery systems.

Paper Code	Relevant Paper Information								Research Questions					Paper Abstract
Id	Paper Title	Authors	Year	Publisher	Research Country	Journal	Number of Pages	Total Citations on Google Scholar	RQ1 – What phase the development of the delivery systems of chitosan/insulin are? (initial,encapsulation, *in vitro* realise, and *in vivo* realise)	RQ2 – What are the release system developed? (Hidrogel, nanoemution, nanoparticles, scaffold, membrene, fiber, nanofiber, film, gel, etc.)	RQ3 – What are the main alternative ways of administration suggested? (Oral, transbuccal, buccal, Nasal,subcutaneous, transdermic, injetable, etc.)	RQ4 – What are the total amount of insulin Loaded and the encapsulation efficiency of the release system? (Encapsulation efficiency and loading capacity)	RQ5 – For how long the system released the insulin? (Toatal time of release)	Abstract
1	Development and characterization of in situ gel system for Nasal insulin delivery	A. K. Agrawal, P. N. Gupta, A. Khanna1, R. K. Sharma, H. K. Chandrabanshi, N. Gupta, U. K. Patil, S. K. Yadav	200 9	Govi-Verlag Pharmazautischer Verlag	India	Pharmazie	6	47	*in vivo*	Hydrogels	Nasal	Not informed	6 h	The objective of the present study was to develop a thermosensitive in situ gel system based on chitosan and poly vinyl alcohol (PVA) for Nasal delivery of insulin. The hydrogel was prepared by mixing chitosan and PVA. The concentration of the components was optimized during formulation development. The prepared hydrogel was characterized for gelation temperature, gelation time, viscosity changes, degree of swelling, in vitro release and in vivo hypoglycemic effect. The prepared hydrogel was liquid at room temperature while underwent thermal transition from solution below or at room temperature to non-flowing hydrogel when incubated at 37 ◦C for approximately 12 minutes with

														increased viscosity. The in vitro release of insulin from gel network was observed spectrophotometrically which was good enough to maintain blood glucose level for six hour. Furthermore, the formulation when evaluated for their in vivo hypoglycemic effect, demonstrated its ability to reduce glucose level. The observed in vitro and in vivo results indicate that the proposed thermosensitive in situ gelling system has substantial potential as Nasal delivery system for insulin.
2	Low Molecular Weight Chitosan–Insulin Polyelectrolyte Complex: Characterization and Stability Studies	Zakieh I. Al-Kurdi, Babur Z. Chowdhry, Stephen A. Leharne, Mahmoud M. H. Al Omari and Adnan A. Badwan	2015	MDPI	Jordan	Marine Drug	20	11	*in vitro*	Nanoparticles	Oral	Not informed	6 h	The aim of the work reported herein was to investigate the effect of various low molecular weight chitosans (LMWCs) on the stability of insulin using USP HPLC methods. Insulin was found to be stable in a polyelectrolyte complex (PEC) consisting of insulin and LMWC in the presence of a Tris-buffer at pH 6.5. In the presence of LMWC, the stability of insulin increased with decreasing molecular weight of LMWC; 13 kDa LMWC was the most efficient molecular weight for enhancing the physical and chemical stability of insulin. Solubilization of insulin-LMWC polyelectrolyte comple+P2x (I-LMWC PEC) in a reverse micelle (RM) system,

														administered to diabetic rats, results in an Oral delivery system for insulin with acceptable bioactivity.
3	Chitosan/lecithin liposomal nanovesicles as na Oral insulin delivery system	Mayyas Al-Remawi, Amani Elsayed, Ibrahim Maghrabi, Mohammad Hamaidi & Nisrein Jaber	2016	Taylor & Francis	Jordan/Saudi Arabia	Pharmaceutical Development and Technology	9	14	*in vivo*	Nanoparticles	Oral	The AE was calculated to be around 20% under such preparation conditions.	1 h	In the present work, insulin–chitosan polyelectrolyte complexes associated to lecithin liposomes were investigated as a new carrier for Oral delivery of insulin. The preparation was characterized in terms of particle size, zeta potential and encapsulation efficiency. Surface tension measurements revealed that insulin–chitosan polyelectrolyte complexes have some degree of hydrophobicity and should be added to lecithin liposomal dispersion and not the vice versa to prevent their adsorption on the surface. Stability of insulin was enhanced when it was associated to liposomes. Significant reduction of blood glucose levels

														was noticed after Oral administration of liposomal preparation to streptozotocin diabetic rats compared to control. The hypoglycemic activity was more prolonged compared to subcutaneously administered insulin.
4	Preparation and characterization of insulin nanoparticles using chitosan and Arabic gum with ionic gelation method	Mohammad Reza Avadi, Assal Mir Mohammad Sadeghi, Nasser Mohammadpour, Pharm, Saideh Abedin, Pharm, Fatemeh Atyabi, Rassoul Dinarvand, Morteza Rafiee-Tehrani	2010	Elsevier	Iran	Nanomedicine	6	231	*in vitro*	Nanoparticles	Oral	The LE for all formulations was calculated and was shown to be between 25% and 35%	4 h	In the past decade, many strategies have been developed to enhance Oral protein delivery. The aim of the current work was to develop a nanoparticulate system based on ionic gelation between chitosan and Arabic gum for loading of insulin. Various formulations were prepared using 23 factorial designs. The optimum association efficiency was obtained for formulations F2, F5, and F8. The release profile of insulin in phosphate buffer solutions (pH 6.5 and pH 7.2) is completely different than that in acidic medium (pH 1.2). Increased solubility of chitosan in acidic medium and better swelling of Arabic gum chains at pH N6.5 resulted in lower insulin release of nanoparticles at pH 6.5 in comparison with that of the other pH mediums. The values of the exponent n were 0.49 and 0.82 for

														formulations F8 and F5, respectively, indicating a non-Fickian transport. This suggests that release is possibly controlled by diffusion or relaxation of the polymer chains.
5	Ultrasound-triggered noninvasive regulation of blood glucose levels using microgels integrated with insulin nanocapsules	Jin Di, Jicheng Yu, Qun Wang, Shanshan Yao, Dingjie Suo, Yanqi Ye, Matthew Pless, Yong Zhu, Yun Jing, and Zhen Gu	201 7	Springer	United States	Nano Research	10	28	*in vivo*	Microgels	Injectable	Drug loading capacity (LC%) and encapsulation efficiency (EE%) of nanoparticles encapsulated with insulin was 11,9 (+/- 0,6) and 71,3 (+/- 1,8), respectivily.	240 h	Diabetes is a serious public health problem affecting 422 million people worldwide. Traditional diabetes management often requires multiple daily insulin injections, associated with pain and inadequate glycemia control. Herein, we have developed an ultrasound-triggered insulin delivery system capable of pulsatile insulin release that can provide both long-term sustained and fast on-demand responses. In this system, insulin-loaded poly(lactic-co-glycolic acid) (PLGA) nanocapsules are encapsulated within chitosan microgels. The encapsulated insulin in nanocapsules can passively diffuse from the nanoparticle but remain restricted within the microgel. Upon ultrasound treatment, the stored insulin in microgels can be rapidly released to

														regulate blood glucose levels. In a chemically-induced type 1 diabetic mouse model, we demonstrated that this system, when activated by 30 s ultrasound administration, could effectively achieve glycemic control for up to one week in a noninvasive, localized, and pulsatile manner.
6	Chitosan–Sodium Lauryl Sulfate Nanoparticles as a Carrier System for the In Vivo Delivery of Oral Insulin	Amani Elsayed, Mayyas Al-Remawi, Nidal Qinna, Asim Farouk, Khaldoun A. Al-Sou'od, and Adnan A. Badwan	2011	Springer	Jordan	AAPS PharmSciTech	7	43	*in vivo*	Nanoparticles	Oral	Nanoparticles displayed high encapsulation efficiency as 82.04±1.95% of insulin was encapsulated.	7 h	The present work explores the possibility of formulating an Oral insulin delivery system using nanoparticulate complexes made from the interaction between biodegradable, natural polymer called chitosan and anionic surfactant called sodium lauryl sulfate (SLS). The interaction between chitosan and SLS was confirmed by Fourier transform infrared spectroscopy. The nanoparticles were prepared by simple gelation method under aqueous-based conditions. The nanoparticles were stable in simulated gastric fluids and could protect the encapsulated insulin from the GIT enzymes. Additionally, the in vivo results clearly indicated that the insulin-loaded nanoparticles could effectively reduce the blood glucose level in a diabetic rat model. However, additional formulation modifications are required to improve

														insulin Oral bioavailability.
7	An integrated buccal delivery system combining chitosan films impregnated with peptide loaded PEG-b-PLA nanoparticles	Concetta Giovino, Isaac Ayensu, John Tetteh, Joshua S. Boateng	2013	Elsevier	United Kingdom	Colloids and Surfaces B: Biointerfaces	7	69	in vivo	films	Buccal	Not informed	360 h	Peptide (insulin) loaded nanoparticles (NPs) have been embedded into buccal chitosan films (Ch-films- NPs). These films were produced by solvent casting and involved incorporating in chitosan gel (1.25% w/v), NPs-Insulin suspensions at three different concentrations (1, 3, and 5mg of NPs per film) using glycerol as plasticiser. Film swelling and mucoadhesion were investigated using 0.01M PBS at 37 ◦C and texture analyzer, respectively. Formulations containing 3mg of NPs per film produced optimised films with excellent mucoadhesion and swelling properties. Dynamic laser scattering measurements showed that the erosion of the chitosan backbone controlled the release of NPs from the films, preceding in vitro drug (insulin) release from

														Ch-films-NPs after 6 h. Modulated release was observed with 70% of encapsulated insulin released after 360 h. The use of chitosan films yielded a 1.8-fold enhancement of ex vivo insulin permeation via EpiOralTM buccal tissue construct relative to the pure drug. Flux and apparent permeation coefficient of 0.1_g/cm2/h and 4×10−2 cm2/h were respectively obtained for insulin released from Chfilms- NPs-3. Circular dichroism and FTIR spectroscopy demonstrated that the conformational structure of the model peptide drug (insulin) released from Ch-films-NPs was preserved during the formulation process.
8	Glucose-Responsive Microgels Integrated with Enzyme Nanocapsules for Closed-Loop Insulin Delivery	Zhen Gu, Tram T. Dang, Minglin Ma, Benjamin C. Tang,Hao Cheng, Shan Jiang, Yizhou Dong, Yunlong Zhang,and Daniel G. Anderson	201 3	ACS Publication	United States	ACS Nano	9	223	*in vivo*	Microgels	Injectable	An optimal insulinloading capacity of 44.6 (2.8% and encapsulation efficiency of 59.7 (3.4% (Supporting Information) were achieved.	4 h	A glucose-responsive closed-loop insulin delivery system represents the ideal treatment of type 1 diabetes mellitus. In this study, we develop uniform Injectable microgels for controlled glucose-responsive release of insulin. Monodisperse microgels (256 (18 µm), consisting of a pH-responsive chitosan matrix, enzyme nanocapsules, and recombinant human insulin, were fabricated through a one-step electrospray procedure. Glucose-specific enzymes were covalently

														encapsulated into the nanocapsules to improve enzymatic stability by protecting from denaturation and immunogenicity as well as to minimize loss due to diffusion from the matrix. The microgel system swelled when subjected to hyperglycemic conditions, as a result of the enzymatic conversion of glucose into gluconic acid and protonation of the chitosan network. Acting as a self-regulating valve system, microgels were adjusted to release insulin at basal release rates under normoglycemic conditions and at higher rates under hyperglycemic conditions. Finally, we demonstrated that these microgels with enzyme nanocapsules facilitate insulin release and result in a reduction of blood glucose levels in a mouse model of type 1 diabetes.

9	Chitosan/cyclodextrin nanoparticles as macromolecular drug delivery system	Alexander H. Krauland, Maria José Alonso	2007	Elsevier	Spain	International Journal of Pharmaceutics	9	202	*in vitro*	Nanoparticles	Nasal	Insulin could be incorporated very efficiently to all nanoparticle formulations, reaching association efficiencies of more than 85% and loading efficiency with 68.4 ± 0.5%	2 h	The aim of this study was to generate a new type of nanoparticles made of chitosan (CS) and carboxymethyl- -cyclodextrin (CM- -CD) and to evaluate their potential for the association and delivery of macromolecular drugs. CS and CM- -CD or mixtures of CM- -CD/tripolyphosphate (TPP) were processed to nanoparticles via the ionotropic gelation technique. The resulting nanoparticles were in the size range of 231–383 nm and showed a positive zeta potential ranging from +20.6 to +39.7mV. These nanoparticles were stable in simulated intestinal fluid pH 6.8 at 37 ◦C for at least 4 h. Elemental analysis studies revealed the actual integration of CM- -CD to CS nanoparticles. Insulin and heparin used as macromolecular model drugs, could be incorporated into the different nanocarriers with association efficiencies of 85.5–93.3 and 69.3–70.6%, respectively. The association of these compounds led to an increase of the size of the nanoparticles (366–613 nm), with no significant modification of their zeta potentials (+23.3 to +37.1 mV). The release profiles of the associated macromolecules were highly dependent on the type of molecule

														and its interaction with the nanomatrix: insulin was very fast released (84–97% insulin within 15 min) whereas heparin remained highly associated to the nanoparticles for several hours (8.3–9.1% heparin within 8 h). In summary, CS-CD (cyclodextrin) nanoparticles may be considered as nanocarriers for the fast or slow delivery of macromolecules.

10	Electrostatic Self-Assembled Chitosan-Pectin Nano- and Microparticles for Insulin Delivery	Vinicius B. V. Maciel, Cristiana M. P. Yoshida, Susana M. S. S. Pereira, Francisco M. Goycoolea and Telma T. Franco	2017	MDPI	Brazil	Molecules	21	13	*in vitro*	Nano- and microparticles	Oral	An EE of 34–37% of insulin was achieved for systems with charge ratio (n+/n−) 0.25. The EE was further improved (62%) for systems with charge ratio (n+/n−) 5.00, independent of DA of chitosan.	2 h	A polyelectrolyte complex system of chitosan-pectin nano- and microparticles was developed to encapsulate the hormone insulin. The aim of this work was to obtain small particles for Oral insulin delivery without chemical crosslinkers based on natural and biodegradable polysaccharides. The nano- and microparticles were developed using chitosans (with different degrees of acetylation: 15.0% and 28.8%) and pectin solutions at various charge ratios (n+/n- given by the chitosan/pectin mass ratio) and total charge. Nano- and microparticles were characterized regarding particle size, zeta potential, production yield, encapsulation efficiency, stability in different media, transmission electron microscopy and cytotoxicity assays using Caco-2 cells. The insulin release was evaluated in vitro in simulated gastric and intestinal media. Small-sized particles (~240–~1900 nm) with a maximum production yield of ~34.0% were obtained. The highest encapsulation efficiency (~62.0%) of the system was observed at a charge ratio (n+/n-) 5.00. The system was stable in various media,

														particularly in simulated gastric fluid (pH 1.2). Transmission electron microscopy (TEM) analysis showed spherical shape particles when insulin was added to the system. In simulated intestinal fluid (pH 6.8), controlled insulin release occurred over 2 h. In vitro tests indicated that the proposed system presents potential as a drug delivery for Oral administration of bioactive peptides.
11	Development and characterization of new insulin containing polysaccharide nanoparticles	Bruno Sarmento, António Ribeiro, Francisco Veiga c, omingos Ferreira	2006	Elsevier	Portugal	Colloids and Surfaces B: Biointerfaces	10	219	*in vitro*	Nanoparticles	Oral	Nanoparticles formulated with a DS:chitosan mass ratio of 1.5:1 showed a AE (%) of 85,4 (+/- 0,5).	5 h	A nanoparticle insulin delivery system was prepared by complexation of dextran sulfate and chitosan in aqueous solution. Parameters of the formulation such as the final mass of polysaccharides, the mass ratio of the two polysaccharides, pH of polysaccharides solution, and insulin theorical loading were identified as the modulating factors of nanoparticle physical properties. Particles with a mean diameter of 500 nm and a zeta potential of approximately −15mV were produced under optimal conditions of DS:chitosan mass ratio of 1.5:1 at pH 4.8. Nanoparticles showed spherical shape,

														uniform size and good shelf-life stability. Polysaccharides complexation was confirmed by differential scanning calorimetry and Fourier transformed infra-red spectroscopy. An association efficiency of 85% was obtained. Insulin release at pH below5.2was almost prevented up to 24 h and at pH 6.8 the release was characterized by a controlled profile. This suggests that release of insulin is ruled by a dissociation mechanism and DS/chitosan nanoparticles are pH-sensitive delivery systems. Furthermore, the released insulin entirely maintained its immunogenic bioactivity evaluated by ELISA, confirming that this new formulation shows promising properties towards the development of an Oral delivery system for insulin.

12	Preparation and characterization of chitosan–polyvinyl alcohol blend hydrogels for the controlled release of nano-insulin	Yuangang Zu, Ying Zhang, Xiuhua Zhao, Chang Shan, Shuchong Zu, Kunlun Wang, Yong Li, Yunlong Ge	201 2	Elsevier	China	International Journal of Biological Macromolecules	6	77	in vitro	Hydrogels	Transdermal	Not informed	12 h	Chitosan (CS)–polyvinyl alcohol (PVA) blend hydrogels were prepared using glutaraldehyde as the crosslinking agent. The obtained hydrogels, which have the advantages of both PVA and CS, can be used as a material for the transdermal drug delivery (TDD) of insulin. The nano-insulin-loaded hydrogels were prepared under the following conditions: 1.2 g of polyethylene glycol, 1.5 g of CS, 1.2 g of PVA, 1.2 mL of 1% glutaraldehyde solution, 16 mL of water, and 40 mg of nano-insulin with 12 min of mixing time and 3 min of cross-linking time. The nano-insulin-loaded hydrogels were characterized using scanning electron microscopy, energy dispersive spectrometry, Fourier-transform infrared spectroscopy, differential scanning calorimetry, thermogravimetric analysis, X-ray diffraction, and its mechanical properties were analyzed. The results show that all molecules in the hydrogel have good compatibility and they formed a honeycomb-like structure. The hydrogel also showed good mechanical and thermal properties. The in vitro drug release of the hydrogel showed that the nano-insulin accorded with Fick's first law of

														diffusion and it has a high permeation rate (4.421 _g/(cm2 h)). These results suggest that the nano-insulin-loaded hydrogels are a promising non-invasive TDD system for diabetes chemotherapy.
13	Microencapsulated chitosan nanoparticles for pulmonary protein delivery: In vivo evaluation of insulin-loaded formulations	S. Al-Qadi, A. Grenha, D. Carrión-Recio, B. Seijo, C. Remuñán-López	2012	Elsevier	Spain	Journal of Controlled Release	8	146	*in vivo*	Nanoparticles	Intratracheal	CS/TPP/INS=5/1/1.5 (w/w), which have higher TPP content, registered increased production yield (48%), INS association efficiency (75%), loading capacity (31%) and zeta potential (+32 mV) compared to the other formulation (6/1/1.8 (w/w)). CS 113 show a higher production yield, association efficiency and loading capacity (56%, 83%, and 37%, respectively), but smaller size	5 h	This work presents a new dry powder system consisting of microencapsulated protein-loaded chitosan nanoparticles (CS NPs). The developed system was evaluated in vivo in rats in order to investigate its potential to transport insulin (INS), a model protein, to the deep lung, where it is absorbed into systemic circulation. The INS-loaded CS NPs were prepared by ionotropic gelation and characterized for morphology, size, zeta potential, association efficiency and loading capacity. Afterwards, the NPs were co-spray dried with mannitol resulting in a dry powder with adequate aerodynamic

												(289 nm) than the NPs made of CS 213.		properties for deposition in deep lungs. The assessment of the plasmatic glucose levels following intratracheal administration to rats revealed that the microencapsulated INS-loaded CS NPs induced a more pronounced and prolonged hypoglycemic effect compared to the controls. Accordingly, the developed system constitutes a promising alternative to systemically deliver therapeutic macromolecules to the lungs, but it can also be used to provide a local effect.
14	A novel nanoemulsion-based method to produce ultrasmall, water-dispersible nanoparticles from chitosan, surface modified with cell-penetrating peptide for Oral delivery of proteins and peptides	Ghullam Reza Barbari, Farid Abedin Dorkoosh, Mohsen Amini, Mohammad Sharifzadeh, Fateme Atyabi, Saeed Balalaie, Niyousha Rafiee Tehrani, Morteza Rafiee Tehrani	2017	Dove Medical Press	Iran	International Journal of Nanomedicine	13	12	*in vitro*	Nanoparticles	Oral	NPs weight ratio of 2:10 with 12 h incubation time represent the highest LE and EE of 16 and 92%, respectively.	24 h	A simple and reproducible water-in-oil (W/O) nanoemulsion technique for making ultrasmall (,15 nm), monodispersed and water-dispersible nanoparticles (NPs) from chitosan (CS) is reported. The nano-sized (50 nm) water pools of the W/O nanoemulsion serve as “nanocontainers and nano-reactors”. The entrapped polymer chains of CS inside these “nano-reactors” are covalently cross-linked with the chains of polyethylene glycol (PEG), leading to rigidification and formation of NPs. These NPs possess excessive swelling properties in aqueous medium and preserve integrity in all pH

														ranges due to chemical cross-linking with PEG. A potent and newly developed cell-penetrating peptide (CPP) is further chemically conjugated to the surface of the NPs, leading to development of a novel peptide-conjugated derivative of CS with profound tight-junction opening properties. The CPP-conjugated NPs can easily be loaded with almost all kinds of proteins, peptides and nucleotides for Oral delivery applications. Feasibility of this nanoparticulate system for Oral delivery of a model peptide (insulin) is investigated in Caco-2 cell line. The cell culture results for translocation of insulin across the cell monolayer are very promising (15%–19% increase), and animal studies are actively under progress and will be published separately.

15	Synthesis of a novel structure for the Oral delivery of insulin and the study of its effect on diabetic rats	Akbar Esmaeilia, Syed Neda Mousavi	2017	Elsevier	Iran	Life Sciences	7	2	*in vivo*	Scaffolds	Oral	Not informed	32 h	Common materials used for drug delivery in the body are: liposomes, micelles, polymer capsules, dendrimers, nanoparticles, porous materials, etc. Drug delivery system should be inert, biodegradable, have high biocompatibility and the ability to load large amounts of the drug with known concentration while having a simple and economical sterilizing process. In this study we produced mesoporous silica nanostructures coated with polyamide amine dendrimer that were placed in chitosan-gelatin scaffolds. At every step of the synthesis, the products were identified using different methods, including XRD, FT-IR, SEM, and TGA. The final drug was studied in terms of in vitro & in vivo and MTT toxicity was evaluated.
16	A cell-penetrating peptide mediated chitosan nanocarriers forimproving intestinal insulin delivery	Lei Li, Liaoqing Yang, Manman Li, Liefeng Zhang	2017	Elsevier	China	Carbohydrate Polymers	8	9	*in vitro*	Nanoparticles	Oral	the encapsulation efficiency and drug loading content of the CS/insulin-NPs were 73.68% and 7.89%, respectively.	4.5 h	To overcome barriers for Oral delivery of insulin, the chitosan(CS)-based nanocarriers with a novel cellpenetrating peptide (SAR6EW) have been prepared and evaluated in this study. Characterization mea-surements showed that SAR6EW/CS/insulin-NPs displayed global particles with smooth surfaces and anaverage diameter about 150 nm. The entrapment efficiency and loading rates of

														insulin were 75.36% and7.58%, respectively. Insulin could be released constantly from SAR6EW/CS/insulin-NPs in vitro. Further-more, SAR6EW/CS/insulin-NPs could facilitate the uptake of insulin and induce a significantly higherinternalization of insulin via adding clathrin and caveolae mediated endocytosis. In addition, in vivohypoglycemic studies showed that Orally administrated SAR6EW/CS/insulin-NPs produced a better hypo-glycemic effect as compared with CS/insulin-NPs in diabetic rats. Meanwhile, no significant cytotoxicityof the nanoparticles was observed. In conclusion, SAR6EW-mediated chitosan nanocarriers showed suf-ficient effectiveness for Oral delivery of insulin. This delivery system is also promising for the delivery ofother protein drugs by Oral administration.

17	Nasal absorption enhancement of insulin using PEG-grafted chitosan nanoparticles	Xinge Zhang, Huijie Zhang, Zhongming Wu, Zhen Wang,Haimei Niu, Chaoxing Li	2008	Elsevier	China	European Journal of Pharmaceutics and Biopharmaceutics	9	157	*in vivo*	Nanoparticles	Nasal	The PEG-g-chitosan nanoparticles displayed a high association efficiency (>78.6%) leading to insulin loading values as high as 38.6%	24 h	The objective of this work was to explore the potential of polyethylene glycol-grafted chitosan (PEG-g-chitosan) nanoparticles as a system for improving the systemic absorption of insulin following Nasal administration. Insulin-loaded PEG-g-chitosan nanoparticles were prepared by the ionotropic gelation of PEG-g-chitosan solution using tripolyphosphate ions as the crosslinking agent. The nanoparticles were in the size range 150–300 nm, had a positive electrical charge (+16 to +30 mV) and were associated with insulin (loading efficiency 20–39%). The physicochemical properties of nanoparticles were affected by the composition of the copolymer. In vitro insulin release studies showed an initial burst followed by a slow release of insulin. IntraNasal administration of PEG-g-chitosan nanoparticles in rabbits enhanced the absorption of insulin by the Nasal mucosa to a greater extent than a suspension of insulin-PEG-g-chitosan and control insulin solution. PEG-g-chitosan nanoparticles are promising vehicles for insulin transport through the Nasal mucosa.

18	The influence of spatial distribution on add-on therapy of designed Ca-Alg/CS MEMs system	Qinglei Dai, Xia Zhou, Kejing Wu, Ruimin Long, Shibin Wang, Haiwang Huang, Yanhua Xia & Yuangang Liu	2018	Taylor & Francis	China	Journal of Biomaterials Science, Polymer Edition	12	0	*in vivo*	Microspheres	Injectable	The drug-loading and the encapsulation efficiency for the two drugs loaded alone were respectively attained a different value (0.204 ± 0.023% (inner) and 0.241 ± 0.017% (outer), 1.641 ± 0.180% (inner) and 1.804 ± 0.121% (outer) were for MET; 2.296 ± 0.120% (inner) and 9.357 ± 0.751% (outer), 11.662 ± 0.708% (inner) and 85.534 ± 1.511% (outer) were for INS).	48 h	To improve the efficacy and reduce the systemic toxicity of the diabetes mellitus, herewith, we developed a novel microparticles-embedded microcapsules (MEMs) system, synthesized from calcium alginate/ chitosan (Ca-Alg/CS), by emulsion gelation using a high voltage electrostatic droplet generator. In our study, we selected two antidiabetic drugs insulin (INS) and metformin (MET) as model drugs to investigate different spatial distribution appropriate of MEMs system. Characterization based on particle size and morphology, encapsulation efficiency and drug loading, as well as drug delivery properties were carried out on the MEMs system. Typical multi-chamber structure was shown by SEM and the optical spectra. The average diameters of microparticles and Ca-Alg/CS MEMs were 2100 nm and 410 µm, respectively. Insulin and MET were embedded into MEMs via electrostatic reaction according to FT-IR spectra. Moreover, drug loading and encapsulation efficiency of INS were higher than that of MET in this system when drugs were loaded alone or together. More

														importantly, this system has potential for orderly drug release and well sustained release when MET in the inner and INS in the outer space could be applied as a combination therapy for diabetes. The obtained in vivo experimental data on diabetes rats has shown that the designed MEMs system resulted in a higher hypoglycemic effect within add-on therapy.
19	Multiboronic acid-conjugated chitosan scaffolds with glucose selectivity to insulin release	Nabil A. Siddiqui, Nashiru Billa & Clive J. Roberts	2017	Taylor & Francis	United Kingdom	Journal of Biomaterials Science, Polymer Edition	14	2	in vitro	Nanoparticles	Not informed	The EE% for FPBAINP and FTBAINP were 56.7% and 57.5% respectively. The LC% for FPBAINP and FTBAINP were calculated to be 45 ± 1.4 mg and 48 ± 1.1 mg of insulin in 100 mg of nanoparticles respectively.	1 h	The principal challenge for the use of boronic acids (BA) as glucose sensors is their lack of specificity for glucose. We examined the selectivity of and insulin release from two boronic acids- (2-formyl-3-thienylboronic acid (FTBA) and 4-formylphenylboronic acid (FPBA)) conjugated chitosan scaffolds to glucose and fructose. Adsorption of glucose to BA: chitosan conjugates was dose-dependent up to 1:1 at 35 and 42% for FPBA and FTBA respectively but the FTBA conjugates adsorbed more glucose and fructose at respective FPBA ratios. The affinity of both BA

														conjugates to glucose decreased with increase in BA ratio. On the other hand, the affinity of both BA conjugates for fructose decreased from ratio 1:1 to 2:1 then rose again at 3:1. Insulin release from FPBA nanoparticles (FPBAINP) and FTBA nanoparticles (FTBAINP) were both concentration-dependent within glyceamically relevant values (1–3 mg/ml glucose and 0.002 mg/ml fructose). Furthermore, the total amounts of insulin released from FPBAINP in both the media were higher than from FTBAINP. Both FPBAINP and FTBAINP have the potential for development as a glucose-selective insulin delivery system in physiological settings.

20	Positive/negative surface charge of chitosan based nanogels and its potential influence on Oral insulin delivery.	Juan Wang, Mengxue Xu, Xiaojie Cheng, Ming Kong, Ya Liu, Chao Feng, Xiguang Chen	2016	Elsevier	China	Carbohydrate Polymers	7	37	in vitro	Nanogels	Oral	Insulin:CMCS/CS-NGs(−) had na EE(%) 73 ± 6.36 and LC(%) 29 ± 3.61, and Insulin:CMCS/CS-NGs(+) had EE(%) 74 ± 8.36 and LC 27 ± 4.04	15 h	To develop insulin delivery system for the treatment of diabetes, two insulin-loaded nanogels with opposite zeta potential (−15.94 ± 0.449 mV for insulin:CMCS/CS-NGs(−) and +17.15 ± 0.492 mV for insulin:CMCS/CS-NGs(+)) were obtained. study, the blood glucose level in insulin:CMCS/CS-NGs(−) group had 3 mmol/L lower than insulin:CMCS/CS-NGs(+) group during 1 h to 11 h after the Oral administration, which demonstrated that negative insulin:CMCS/CS-NGs had a better management of blood glucose than positive ones. 0.449 mV for insulin:CMCS/CS-NGs(−) and +17.15 ± 0.492 mV for insulin:CMCS/CS-NGs(+)) were obtained. Ex vivo results showed that the nanogels with opposite surface charge exhibited different adhesion and permeation in specific intestinal segments. There was no significant differences in adhesion and permeation in rat duodenum, but in rat jejunum, insulin:CMCS/CS-NGs(−) exhibited enhanced adhesion and permeation, which were about 3 folds (adhesion) and 1.7 folds (permeation) higher than insulin:CMCS/CS-NGs(+). These results

														demonstrated that the surface charge property of nanogels determined the absorption sites of CMCS/CS-NGs in small intestine. In vivo study, the blood glucose level in insulin:CMCS/CS-NGs(−) group had 3 mmol/L lower than insulin:CMCS/CS-NGs(+) group during 1 h to 11 h after the Oral administration, which demonstrated that negative insulin:CMCS/CS-NGs had a better management of blood glucose than positive ones.
21	In vitro and in vivo evaluation of thermosensitive chitosan hydrogel for sustained release of insulin.	Farzaneh Ghasemi Tahrir, Fariba Ganji, Ali Reza Mani, and Elham Khodaverdi	2014	Taylor & Francis	Iran	Drug Delivery	9	19	In vivo	Hydrogels	Injectable	Not informed	>150 h	Injectable In situ gel-forming chitosan/b-glycerol phosphate (CS/b-Gp) solution can be introduced into the body in a minimally invasive manner prior to solidifying within the target tissue. This hydrogel is a good candidate for achieving a prolonged drug delivery system for insulin considering its high molecular weight. In addition to the physicochemical characterization of this hydrogel, in vitro and in vivo applications were studied as a sustained insulin delivery system. In the in vitro release studies, 19–63% of total insulin was released from the CS/b-Gp hydrogel

														within 150 h at different b-Gp and insulin concentrations. The best formulation was selected for in vivo experimentation to control the plasma glucose of diabetic mice models. The hypoglycemic effect of this formulation following subcutaneous injection in diabetic mice lasted 5 d, significantly longer than that of free insulin solution which lasted several hours
22	Development and evaluation of chitosan and chitosan derivative nanoparticles containing insulin for Oral administration.	Hecq J, Siepmann F, Siepmann J, Amighi K, Goole J.	2015	Taylor & Francis	Belgium/France	Drug Development and Industrial Pharmacy	8	16	In vitro	Nanoparticles	Oral	The most promising formulation (F8) was based on HTCC-33% and the EE was 52 ± 3%.	3,33h	Chitosan and chitosan derivative-based nanoparticles loaded with insulin were prepared by self-assembly, via electrostatic interactions between the negatively charged drug and the positively charged polymers. In the investigated chitosan derivatives, the amine groups were substituted to different extents (33, 52 or 99%) by 2-hydroxypropyl-3-trimethyl ammonium groups, rendering the polymers permanently positively charged, irrespective of the pH. This is an important property for this type of advanced drug delivery system, since the pH value changes throughout the gastrointestinal tract and electrostatic interactions are of crucial importance for the stability of the nanoparticles. Permanent positive charges are also in favor of

														mucoadhesion. In contrast, the electric charges of chitosan molecules depend on the pH of the surrounding medium. Since the solubility of the chitosan derivatives increased due to the introduction of quaternary ammonium groups, sodium tripolyphosphate (TPP) was added to the systems to create supplementary cross-links and stabilize the nanoparticles. The presence of TPP influenced both the dissolution of the polymer matrix as well as the resulting release kinetics. The underlying drug release mechanisms were found to be more complex than simple diffusion under constant conditions, likely involving also ionic interactions and matrix dissolution. The most promising formulation was based on a chitosan derivative with 33% substitution degree and characterized by a Z-average of 142 ± 10 nm, a zeta potential of 29 ± 1 mV, an encapsulation efficacy of 52 ± 3% and, most importantly, the release of insulin was sustained for more than 210 min.

23	Bioadhesive polysaccharide in protein delivery system: chitosan nanoparticles improve the intestinal absorption of insulin in vivo	Yan Pan, Ying-jian Li, Hui-ying Zhao, Jun-min Zheng, Hui Xu, Gang Wei, Jin-song Hao, Fu-de Cui	2002	Elsevier	China	International Journal of Pharmaceutics	8	666	In vivo	Nanoparticles	Oral	association efficiency and loading capacity of the nanoparticles were affected by the insulin concentration in the TPP solution and the amount of insulin incorporated, with increasing amount ratio of insulin to chitosan leading to a slight decrease of association efficiency and an enhancement of loading capacity (Table 1).	40 h	There are many ongoing investigations to improve the Oral bioavailability of peptide and protein formulations. Bioadhesive polysaccharide chitosan nanoparticles (CS-NPs) would seem to further enhance intestinal absorption of them. In this study, Insulin-loaded CS-NPs were prepared by ionotropic gelation of CS with tripolyphosphate anions. Its particle size distribution and zeta potential were determined by photon correction spectroscopy and laser Dopper anemometry. The ability of CS-NPs to enhance intestinal absorption of insulin and increase the relative pharmacological bioavailability of insulin was investigated by monitoring the plasma glucose level of alloxan-induced diabetic rats after Oral administration of various doses of insulin-loaded CS-NPs. CS-NPs had a particle size in the range of 250/400 nm and its polydispersity index was smaller than 0.1, positively charged, stable. Insulin association was found up to 80% and its in vitro release showed a great initial burst with a pH-sensitivity property. CS-NPs enhanced the intestinal absorption of insulin to a greater

														extent than the aqueous solution of CS in vivo. Above all, after administration of 21 I.U./kg insulin in the CS-NPs, the hypoglycemia was prolonged over 15 h and the average pharmacological bioavailability relative to SC injection of insulin solution was up to 14.9%.
24	[Oral insulin delivery by self-assembled chitosan nanoparticles: In vitro and in vivostudies in diabetic animal model]()	Piyasi Mukhopadhyay, Kishor Sarkar, Mousumi Chakraborty, Sourav Bhattacharya, Roshnara Mishra, P.P. Kundu	2013	Elsevier	India	Materials Science and Engineering: C	6	81	In vivo	Nanoparticles	Oral	~≈ 97% insulin encapsulation and 27% insulin loading capacity.	12 h	We have developed self-assembled chitosan/insulin nanoparticles for successful Oral insulin delivery. The main purpose of our study is to prepare chitosan/insulin nanoparticles by self-assembly method, to characterize them and to evaluate their efficiency in vivo diabetic model. The size and morphology of the nanoparticles were analyzed by dynamic light scattering (DLS), atomic force microscopy (AFM) and scanning electron microscopy (SEM). The average particle size ranged from 200 to 550 nm, with almost spherical or sub spherical shape. An

														average insulin encapsulation within the nanoparticles was ~85%. In vitro release study showed that the nanoparticles were also efficient in retaining good amount of insulin in simulated gastric condition, while significant amount of insulin release was noticed in simulated intestinal condition. The Oral administrations of chitosan/insulin nanoparticles were effective in lowering the blood glucose level of alloxan-induced diabetic mice. Thus, self-assembled chitosan/insulin nanoparticles show promising effects as potential insulin carrier system in animal models
25	A novel approach to Oral delivery of insulin by conjugating with low molecular weight chitosan.	Lee E, Lee J, Jon S.	2010	ACS Publication	Korea	Biocomjugate Chemistry	3	53	In vivo	Not informed (Conjugate)	Oral	Not informed	12 h	new Oral delivery system for insulin was developed aiming to improve bioavailability based on a conjugate between insulin and low molecular weight chitosan (LMWC) of narrow molecular weight distribution. The conjugate was synthesized from the reaction between site-specifically modified insulin at the lysine residue of the B-chain and sulfhydryl-modified LMWC. To investigate the effect of MWs of LMWC on Oral bioavailability of insulin, various LMWCs (3, 6, 9, and 13k average MW) with narrow MW distribution were used to synthesize LMWC-

														insulin conjugates. The content of insulin in the LMWC-insulin conjugates was calculated by UV spectrophotometer: 62%, 44%, 38%, and 29% for 3, 6, 9, and 13 kDa LMWC, respectively. The biological activity of insulin in LMWC(6k)-insulin conjugate in vivo was 43 (0.7%. LMWC-insulin conjugates after Oral administration to diabetic rat models could control blood glucose levels effectively for several hours. Of those conjugates, LMWC(9k)-insulin exhibited the highest pharmacodynamic bioavailability of 3.7 (0.3% relative to that of subcutaneously (s.c.) injected insulin (100%).
26	In Vitro Insulin Release from Thermosensitive Chitosan Hydrogel	Elham Khodaverdi, Mohsen Tafaghodi,Fariba Ganji, Khalil Abnoos, and Hanie Naghizadeh	2012	Springer	Iran	American Association of Pharmaceutical Scientists	6	63	In vitro	Hydrogels	Injectable	Not informed	>300h	Recently, great attention has been paid to in situ gel-forming chitosan/glycerol-phosphate (chitosan/Gp) solution due to their good biodegradability and thermosensitivity. This in situ gel-forming system is Injectable fluid that can be introduced into the body in a minimally invasive manner prior to solidifying within the desired tissue. At the present study, insulin release from chitosan/Gp solution has been investigated. Insulin in different concentrations was loaded in two

														formulations of chitosan/Gp solution and in vitro drug release was studied over a period of 3 weeks. Results indicated that the release of insulin from chitosan/Gp gel decreases by increasing in Gp salt and initial insulin concentration. Stability of released insulin was investigated by 8-anilino-1-naphthalenesulfonate probe. Results proved that insulin have been released in its native form. Because of simple preparation and administration, prolonged release of insulin and stability of released insulin, this in situ gel-forming system could be used as a controlled release delivery system for insulin
27	Design and evaluation of biodegradable, biosensitive in situ gelling system for pulsatile delivery of insulin	Kashyap N, Viswanad B, Sharma G, Bhardwaj V, Ramarao P, Ravi Kumar MN.	2007	Elsevier	India	Biomaterials	9	118	In vivo	Gels	Injectable	Not informed	30h	Biodegradable glucose-sensitive in situ gelling system based on chitosan for pulsatile delivery of insulin was developed. The sols/gels were thoroughly characterized for swelling properties, rheology, texture analysis and water content. The developed glucose-sensitive gels responded to varied glucose concentrations in vitro indicating their ability to function as environment-sensitive systems. Insulin load onto the gels was optimized and was found to affect the rheological behavior of

														these gels, the final preparation used for in vitro contained 1 IU/200 ml of the sol. These gels released the entrapped insulin in a pulsatile manner in response to the glucose concentration in vitro. Furthermore, the formulations when evaluated for their in vivo efficacy in streptozotocin-induced diabetic rats at a dose of 3 IU/kg, demonstrated their ability to release insulin in response to glucose concentration and were preferred much better against subcutaneously given plain insulin formulation used as the control. Together, these preliminary results indicate that biosensitive chitosan in situ gelling systems have substantial potential as pulsatile delivery systems for insulin.

28	Predictive modeling of insulin release profile from cross-linked chitosan microspheres	S. Jose, J.F. Fangueiro, J. Smitha, T.A. Cinu, A.J. Chacko, K. Premaletha, E.B. Souto	2013	Elsevier	India/Portugal	European Journal of Medicinal Chemistry	4	55	In vitro	Microspheres	Oral	Not informed	12 h	Insulin-loaded microspheres composed of chitosan 3% (w/v), and loading 120 IU insulin were produced by emulsion cross-linking method. Cross-linking time was 5 h and glutaraldehyde 3.5% (v/v) was used as cross-linker. Swelling ratio studies were evaluated to predict release of insulin from chitosan microspheres. Bacitracin and sodium taurocholate were incorporated in the formulations as proteolytic enzyme inhibitor and absorption enhancer, respectively. In vitro insulin release studies were performed in phosphate buffer pH 7.4 and also in HCl pH 2 with and without trypsin. Activity of bacitracin was also evaluated. In vitro release showed a controlled profile up to 12 h and the formulation containing 0.15% (w/v) of bacitracin revealed a maximum biological activity of about 49.1 4.1%. Mathematical modeling using Higuchi and KorsmeyerePeppas suggested a non-Fickian diffusion as the mechanism of insulin release. Insulin-loaded chitosan microspheres for Oral delivery showed to be an innovative and reliable delivery system to overcome conventional insulin therapy

29	Preparation and Characterization of Water-Soluble Chitosan Microparticles Loaded with Insulin Using the Polyelectrolyte Complexation Method	Sihui Wu, Yi Tao,Hongliang Zhang,and Zhengquan Su	2011	ACM DL	China	Jornal of Nanomaterials	6	10	In vitro	Microparticles	Oral	Association efficiency and loading capacity of insulin-loaded WSC-MPs prepared in 0.01 mol/L HCl of insulin were 48.28 ± 0.90% and 9.52 ± 1.34%.	24h	Polymeric delivery systems based on microparticles have emerged as a promising approach for perOral insulin delivery. The amount of insulin was quantified by the improved Bradford method. It was shown that water-soluble chitosan/insulin/tripolyphosphate (TPP) mass ratio played an important role in microparticles formation. Stable, uniform, and spherical water-soluble chitosan microparticles (WSC-MPs) with high insulin association efficiency were formed at or close to optimized WSC/insulin/TPP mass ratio. WSC-MPs had higher association efficiency in the pH 4.0 and pH 9.7 of TPP solution. The results showed that association efficiency and loading capacity of insulin-loaded WSC-MPs prepared in 0.01 mol/L HCl of insulin were 48.28 ± 0.90% and 9.52 ± 1.34%. The average size of insulin-loaded WSC-MPs was 292 nm. The presented WSC microparticulate system has promising properties towards the development of an Oral delivery system for insulin.
30	Properties of Insulin–Chitosan Complexes Obtained by an Alkylation	Emmanuel Robles, Josué Juérez, María. G. Burboa, Luis E. Gutierrez, Pablo Taboada, Vıctor	2013	Wiley Online Library	México/Spain	Journal of Applied Polymer Science	10	10	Initial	Gels	Not informed	Not informed	not informed	In this study, we investigated the influence of hydrophobized chitosan on the formation and thermodynamic and

	Reaction on Chitosan	Mosquera, Miguel A. Valdez												surface tension properties of insulin–chitosan (I–Ch) polyelectrolyte complexes (PECs). We used an alkylation procedure to insert 12 carbon chains along the chitosan macromolecule with final substitution degrees of 5, 10, and 50%. NMR and IR spectroscopy were used to evaluate the success and extent of the hydrophobization procedure. Isothermal titration calorimetry (ITC) was used to determine the type and extent of the existing intermolecular interactions between the different constituting components of the insulin–hydrophobized chitosan PECs. Through the surface tension and diffusion coefficients at the air–water interface and ITC experiments with different I–Ch proportions, we demonstrated that around 34, 24, 25, and 60–80 insulin molecules saturated 0, 5, 10, and 50% hydrophobized chitosans, respectively. Surface tension experiments at the air–water interface demonstrated that the interaction of insulin molecules on the unmodified chitosan increased the hydrophobicity; this was mainly due to electrostatic interaction. On the contrary, insulin–hydrophobized

														chitosan interaction lowered the PEC hydrophobicity because of insulin alkyl chain interaction, and therefore, the hydrophilic insulin groups at the PEC surface contributed to a higher surface tension.
31	Drug Delivery System Using Biodegradable Nanoparticles Carrier	Do Hun Lee, Ik Joong Kang	2006	J-Stage 20th	Korea	KONA Powder and Particle Journal	7	5	In vivo	Nanoparticles	Transdermal	Not informed	24h	Recently, many biochemists have identified that chitosan is not rejected by the body and that it can improve the effective and safe delivery of drugs and vaccines with its absorptive power. Also, it has been known that chitosan is suitable for controlled drug release thanks to its advantages of biodegradability and bio-compatibility. As the interest into the extension of human life and personal health has been increased, the pharmaceutical and medical worlds have been making efforts to develop more sustained and effective drug release property in a body. This study investigated the individual drug characteristics and drug release behavior by manufacturing the chitosan patch using insulin, a drug used for treating diabetes, at a low temperature, and further tried to find the optimal condition by adding the skin activating agent to the chitosan patch using NOD (Non Obese

														Diabetic) mice. According to the analysis using the chitosan-insulin drug and the skin-activating agent, a dramatic decrease in the blood glucose level was achieved. An experiment was performed in vivo by utilizing chitosan nanoparticles as a biopolymer to control the drug delivery rate at an optimal concentration, pH and temperature. It was also observed that the experiment of the drug delivery by nanoparticles containing insulin could effectively lower the blood glucose of the mouse.

32	Nanoencapsulated chitosan nanoparticles in emulsion-based Oral delivery system: In vitro and in vivo evaluation of insulin loaded formulation	Gülsah Erel, Mustafa Kotmakçı, Hasan Akbaba, Sumru Sozer Karadaglı, Ayse Gülten Kantarcı	2016	Elsevier	Turkey	Journal of Drug Delivery Science and Technology	6	16	In vivo	Nanoparticles	Oral	Not informed	24h	In this study, we aimed to develop a novel protein - nanoencapsulated system for Oral administration. For this purpose, insulin was selected as the model drug. Insulin loaded chitosan nanoparticles (INS-CS-NPs) were obtained by ionic gelation between chitosan (CS) and sodium tripolyphosphate (TPP). Afterwards, as a novel strategy the nanoparticles were loaded into the inner phase of prepared water in oil microemulsion to provide sustained released, increased in vivo stability and enhanced drug absorption in the gastrointestinal tract. By this way, INS-CS-NPs encapsulated in microemulsion (INS-CS-NP-ME) was formed. The in vitro release properties of formulations with different INS:CS and CS:TPP ratios were investigated. In vitro release study in pH 2.5 revealed that insulin release was significantly low under higher CS ratios ($p < 0.05$). Circular dichroism analyses showed that the conformational stability of insulin was not affected from preparation process. Furthermore, in vivo experiments in Wistar Albino rat model demonstrate that INS-CS-NP-ME effectively reduced blood glucose

														levels over a period of 8 h after Oral administration. Based on these findings, we propose that the developed INS-CS-NP-ME system can be a promising alternative dosage form for Oral protein delivery
33	Nasal Delivery of Insulin Using Novel Chitosan Based Formulations: A Comparative Study in Two Animal Models between Simple Chitosan Formulations and Chitosan Nanoparticles	A. M. Dyer,M. Hinchcliffe,P. Watts, J. Castile, I. Jabbal-Gill,R. Nankervis,A. Smith, and L. Illum	2002	Springer	United Kingdom	Pharmaceutical Research	10	246	In vivo	Nanoparticles	Nasal/Subcutaneous	Not informed	5h	Purpose. To investigate whether the widely accepted advantages associated with the use of chitosan as a nasal drug delivery system, might be further improved by application of chitosan formulated as nanoparticles. Methods. Insulin-chitosan nanoparticles

														were prepared by the ionotropic gelation of chitosan glutamate and tripolyphosphate pentasodium and by simple complexation of insulin and chitosan. The nasal absorption of insulin after administration in chitosan nanoparticle formulations and in chitosan solution and powder formulations was evaluated in anaesthetised rats and/or in conscious sheep. Results. Insulin-chitosan nanoparticle formulations produced a pharmacological response in the two animal models, although in both cases the response in terms of lowering the blood glucose levels was less (to 52.9 or 59.7% of basal level in the rat, 72.6% in the sheep) than that of the nasal insulin chitosan solution formulation (40.1% in the rat, 53.0% in the sheep). The insulin-chitosan solution formulation was found to be significantly more effective than the complex and nanoparticle formulations. The hypoglycaemic response of the rat to the administration of post-loaded insulin-chitosan nanoparticles and insulin-loaded chitosan nanoparticles was comparable. As shown in the sheep model, the most effective chitosan formulation for nasal insulin absorption was a chitosan powder

														delivery system with a bioavailability of 17.0% as compared to 1.3% and 3.6% for the chitosan nanoparticles and chitosan solution formulations, respectively. Conclusion. It was shown conclusively that chitosan nanoparticles did not improve the absorption enhancing effect of chitosan in solution or powder form and that chitosan powder was the most effective formulation for nasal delivery of insulin in the sheep mode.
34	Multifunctional Polyelectrolyte Microparticles for Oral Insulin Delivery	Nadezhda G. Balabushevich, Mikhail A. Pechenkin, Elena D. Shibanova,Dmitry V. Volodkin, Elena V. Mikhalchik	2013	Wiley Online Library	Russia/Germany	Macromolecular Bioscience	9	42	In vivo	Microparticles	Oral	The protein encapsulation efficiency was 62–65% for both insulin and BBI. The microparticles were characterized by a high insulin content (~55%).	8h	Multicomponent insulin-containing microparticles are prepared by layer-by-layer assembly of dextran sulfate and chitosan on the core of protein-polyanion complex with or without protease inhibitors. Oral bioavailability of the encapsulated insulin is improved due to the cumulative effect of each component. A physico-chemical study shows that the particle design allows adjustment of the pH-dependent profile of the insulin release, as well as mucoadhesive properties and Ca2þ binding ability of the microparticles. Supplementing the microparticles with 2–3% protease inhibitors fully prevents proteolysis of human insulin. The pharmacological effect of microencapsulated

														insulin in doses 50–100 IU kg1 is demonstrated in chronic experiments after Oral administration to diabetic rats fed ad libitum.
35	Chitosan Nanofibers for Transbuccal Insulin Delivery	Michael G. Lancina, Roopa Kanakatti Shankar, Hu Yang	201 7	wiley Online Library	United States	Journal of Biomedical Materials Research Part A	7	9	In vitro	Nanofibers	Transbuccal	Not informed	24h	n this work, they aimed at producing chitosan based nanofiber mats capable of delivering insulin via the buccal mucosa. Chitosan was electrospun into nanofibers using poly(ethylene oxide) (PEO) as a carrier molecule in various feed ratios. The mechanical properties and degradation kinetics of the fibers were measured. Insulin release rates were determined in vitro using an ELISA assay. The bioactivity of released insulin was measured in terms of Akt activation in pre-adipocytes. Insulin permeation across the

														buccal mucosa was measured in an ex-vivo porcine transbuccal model. Fiber morphology, mechanical properties, and in vitro stability were dependent on PEO feed ratio. Lower PEO content blends produced smaller diameter fibers with significantly faster insulin release kinetics. Insulin showed no reduction in bioactivity due to electrospinning. Buccal permeation of insulin facilitated by high chitosan content blends was significantly higher than that of free insulin. Taken together, the work demonstrates that chitosan-based nanofibers have the potential to serve as a transbuccal insulin delivery vehicle.
36	Factors Involved in Formulation of Oily Delivery System for Proteins Based on PEG-8 Caprylic/Capric Glycerides and Polyglyceryl-6 Dioleate in a Mixture of Oleic Acid with Chitosan	Assaf, Shereen M.; Al-Jbour, Nawzat D.; Eftaiha, Ala'a F.; Elsayed, Amani M.; Al-Remawi, Mayyas M.; Qinna, Nidal A.; Chowdhry, Babur; Leharne, Stephen; Badwan, Adnan A.	2011	Taylor & Francis	Jordan/Kingdom of Saudi Arabia/United Kingdom	Journal of Dispersion Science and Technology	12	12	In vivo	Water/Oil Microemulsion	Oral	Not informed	24 hours	Systematic experimental work is required to improve knowledge related to the use of oily delivery systems. This work aimed to examine the influence of different molecular weights chitosan on formation and solubilization ability of w/o system of Labrasol, Plurol Oleique, water and oleic acid. Phase diagrams were constructed. Size measurements were performed for each surfactant in oleic acid. Interfacial tension of chitosan was measured between oleic acid and water at pH 1.5 and 6.25. Effect

														of chitosan on microemulsion size was studied. When used to deliver rh-insulin to diabetic rats, the mixture showed reduction in blood glucose compared to control.
37	Basic studies on bioadhesive delivery systems for peptide and protein drugs	Andreas Bernkop-Schnürch, Claudia Humenberger, Claudia Valenta	1998	Elsevier	Austria	International Journal of Pharmaceutics	9	71	Initial	Tablets	Peroral	Not informed	12h	We have been evaluating the influence of different drying methods and of ionic crosslinkers on adhesive strength, cohesiveness as well as release behaviour of bioadhesive polymers. Chitosan-EDTA and carbomer were ionically crosslinked via 1,8-diaminooctane or L-lysine. The resulting polymers were either lyophilised or precipitated in acetone and air-dried. Tablets made of these pre-treated polymers (66.7%), mannitol (30%), and the model drug insulin (3.3%) were investigated in vitro. Whereas tablets containing the precipitated and air-dried chitosan-EDTA or carbomer exhibited under our experimental conditions an adhesive strength of 93.2915.6

														and 93.1917.3 mN, it was determined to be 57.799.5 and 56.196.7 mN (mean9S.D.; n=5) for tablets of the same but lyophilised polymers, respectively. The use of ionic crosslinkers led also to a significant reduction in the bioadhesiveness of the dosage form. Furthermore, the stability of tablets could be strongly increased by using ionic crosslinkers and:or the precipitated and air-dried form of chitosan-EDTA or carbomer. Due to the use of ionic crosslinkers, the release rate of insulin was strongly reduced. The results represent helpful basic information for the development of peroral (poly)peptide delivery systems based on bioadhesive polymers.
38	Polyurethane-incorporated chitosan/alginate core-shell nano-particles forcontrolled Oral insulin delivery	Bhattacharyya, Aditi; Nasim, Farhat; Mishra, Roshnara; Bharti, Ram P.; Kundu, P.P.	2018	Wiley Online Library	India	Journal of Applied Polymer Science	16	2	In vivo	Nanoparticles	Oral	The insulin encapsulation efficiencies of CS-ALG, PU-CS/ALG, CS/PU-ALG, and PU-CS/PU-ALG nanoparticles was 58, 79.5, 74.97, and 98.5%, respectively.	about 800 minutes	Chitosan (CS) and polyurethane-chitosan (PU-CS) nano-particles (NPs) were prepared for the core formation by complex coacervation method whereas alginate (ALG) and PU-ALG were crosslinked by ionic gelation method to form the protective shelllayer over the core. Effects of PU incorporation either within the core or shell or both were investigated by different in vitro and in vivo parameters. Fourier transform infrared (FTIR) spectroscopy of

														different compositions of nano-particles showed distinct characteristic peaks for CS, PU, and ALG, indicating their presence in variable ratios. Significance of polyurethane-incorporated systems towards insulin encapsulation efficiency, swelling parameters, insulin release, and in vivo pharmacological effect were also studied. Particle sizes, zeta potential, morphological analysis, mucoadhesion study, and in vivo acute toxicity studies of these core–shell nanoparticles were also performed. Bioavailability of insulin ranged from 9.04 to 11.6% for polyurethane-incorporated chitosan-alginate core–shell nano-particle formulations which was significantly higher than the insulin bioavailability of basic CS/ALG core–shell nanoparticle system.

39	Noninvasive imaging Oral absorption of insulin delivered by nanoparticles and its stimulated glucose utilization in controlling postprandial hyperglycemia during OGTT in diabetic rats.	Chuang EY ; Lin KJ ; Su FY ; Mi FL ; Maiti B ; Chen CT ; Wey SP ; Yen TC ; Juang JH ; Sung HW	2013	Elsevier	Taiwan	Journal of Controlled Release	10	36	In vivo	Nanoparticles	Oral	Their insulin loading efficiency and content were 77.4 ± 3.9% and 17.8 ± 2.4%, respectively.	10 hours	This work examined the feasibility of preparing a pH-responsive nanoparticle (NP) system composed of chitosan and poly(gamma-glutamic acid) conjugated with ethylene glycol tetraacetic acid (gammaPGA-EGTA) for Oral insulin delivery in diabetic rats during an Oral glucose tolerance test (OGTT). OGTT has been used largely as a model to mimic the period that comprises and follows a meal, which is often associated with postprandial hyperglycemia. Based on Forster resonance energy transfer (FRET), this work also demonstrated the ability of gammaPGA-EGTA to protect insulin from an intestinal proteolytic attack in living rats, owing to its ability to deprive the environmental calcium. Additionally, EGTA-conjugated NPs were effective in disrupting the epithelial tight junctions, consequently facilitating the paracellular permeation of insulin throughout the entire small intestine. Moreover, results of positron emission tomography and computer tomography demonstrated the effective absorption of the permeated insulin into the systemic circulation as well as promotion of the

														glucose utilization in the myocardium, and skeletal muscles of the chest wall, forelimbs and hindlimbs, resulting in a significant glucose-lowering effect. Above results indicate that as-prepared EGTA-conjugated NPs are a promising Oral insulin delivery system to control postprandial hyperglycemia and thus may potentially prevent the related diabetic complications.
40	Microcapsules of alginate/chitosan containing magnetic nanoparticles for controlled release of insulin	Priscilla Vanessa Finotelli; Daniel Da Silva; Mauro Sola-Penna; Alexandre Malta Rossi; Marcos Farina; Leonardo Rodrigues Andrade; Armando Yoshihaki Takeuchi; Maria Helena Rocha-Leão	2010	Elsevier	Brazil	Colloids and Surfaces B: Biointerfaces	6	114	In vivo	Microcapsules	Subcutaneous	The insulin encapsulation efficiency was 33.3 ± 5.2% and 34.0 ± 5.0% for alginate and alginate/chitosan beads, for insulin concentration of 10 wt%, respectively.	24 hours	The challenge of this work was to investigate the potential of alginate/chitosan beads containing magnetite nanoparticles as a drug delivery system. The insulin beads were prepared by dripping a solution of sodium alginate containing insulin into a $CaCl_2$ solution. Magnetite nanoparticles of 5nm mean size were synthesized inside the alginate egg-box structure by co-precipitation of Fe(III) and Fe(II) in the presence of NH_4OH. Quantitative analysis revealed that insulin encapsulation depends on the initial protein

														content and 35% of insulin was entrapped by alginate beads for a protein concentration of 10wt%. It was verified that approximately 50% of the insulin was released to Milli-Q water in 800h release experiments. The application of oscillating magnetic field increased three fold the insulin release. The results suggest that the alginate/chitosan system containing magnetite nanoparticles is a promising system for clinical applications of controlled release of insulin in the presence of an oscillating magnetic field in a subcutaneous implant approach.
41	Chitosan-alginate blended nanoparticles as carriers for the transmucosal delivery of macromolecules	Goycoolea, Francisco M.; Lollo, Giovanna; Remunan-Lopez, Carmen; Quaglia, Fabiana; Alonso, Maria J.	2009	ACS Publication	Spain/ Mexico/ Italy	Biomacromolecules	8	176	In vivo	Nanoparticles	Nasal	CS-TPP-ALGnanoparticles were able to associate insulin with efficiencies of between ~41 to ~52% and load efficiency of ~51 to ~53%.	5 hours	Nanoparticles intended for use in the transmucosal delivery of macromolecules were prepared by the ionic gelation of chitosan (CS) hydrochloride with pentasodium tripolyphosphate (TPP) and concomitant complexation with sodium alginate (ALG). The incorporation of a small proportion of ALG of increasing molecular weight (Mw; from 4 to 74 kDa) into the nanoparticles led to a monotonic increase in colloidal size from ~260 to ~525 nm. This increase in size was regarded as a consequence of the formation of gradually

														more expanded structures. Insulin, taken as a model peptide, was associated to CS-TPP-ALG nanoparticles with efficiencies in the range of ~41 to ~52%, irrespective of the Mw of the ALG incorporated in the formulation. These CS-TPP-ALG nanoparticles exhibited a capacity to enhance the systemic absorption of insulin after Nasal administration to conscious rabbits. Interestingly, it was observed that the duration of the hypoglycaemic response was affected by the ALG's Mw. Briefly, this work describes a new nanoparticulate composition of potential value for increasing Nasal insulin absorption.
42	Fabrication and characterization of complex nanoparticles based on carboxymethyl short chain amylose and chitosan by ionic gelation	Ji, Na and Hong, Yan and Gu, Zhengbiao and Cheng, Li and Li, Zhaofeng and Li, Caiming	2018	Royal Society of Chimestry	China	Food & Function	38	2	in vitro	Nanoparticles	Oral	CMSCA/CS NPs show 24 an insulin encapsulation efficiency of 85.2% and loading capacity of 6.55%.	8 hours	We aimed to investigate whether the combination of the modification of short chain amylose (SCA) with chitosan (CS) through the electrostatic interaction could be considered as a candidate for Oral delivery of bioactive ingredients. Carboxymethyl short chain amylose (CMSCA) was synthesized by reacting SCA with monochloroacetic acid. The changes in SCA levels after the reaction were investigated by zeta-potential

														determination, Fourier transform infrared (FTIR) spectroscopy, differential scanning calorimetry, thermogravimetry and derivative thermogravimetry. Complex nanoparticles (NPs) were then synthesized using CMSCA and CS by ionic gelation. FTIR spectral analysis revealed that the complex NPs were synthesized by hydrogen bonding and electrostatic interactions between CMSCA and CS. CMSCA/CS NPs show an insulin encapsulation efficiency of 85.2% and exhibit sustained release of insulin in vitro. CMSCA/CS NPs were observed to show excellent cytocompatibility by cell culture. These findings demonstrated that CMSCA/CS NPs constructed by the ionic gelation method could be further exploited as a potential Oral delivery system for peptide drugs.<br>© 2018 The Royal Society of Chemistry.

43	Characterization of thermosensitive chitosan-based hydrogels by rheology and electron paramagnetic resonance spectroscopy	Sabine Kempe, Hendrik Metz, Martin Bastrop, Annette Hvilsom, Renata Vidor Contri, Karsten Mäder	2008	Elsevier	Germany	European Journal of Pharmaceutics and Biopharmaceutics	8	77	In vitro	Hydrogels	Not informed	Not informed	48 hours	Chitosan, an amino-polysaccharide, has been proposed as a promising biopolymer for tissue repair and drug delivery. Chitosan solutions containing glycerol-2-phosphate (b-GP) have been described as injectable in situ gelling thermosensitive formulations, which undergo sol–gel transition at physiological pH and temperatures. This feature makes them suitable for the parenteral administration of drugs, especially for peptides and proteins. The aim of the present study was to get a deeper insight into the macro- and microstructure of chitosan/b-GP systems. In addition to oscillating rheology, electron paramagnetic resonance (EPR) spectroscopy was applied to examine the microviscosity and pH inside the gels depending on the b-GP concentration and to follow the loading and release of spin-labelled Insulin. All chitosan/b-GP solutions showed a physiological pH ranging from 6.6 to 6.8 that did not change during gelation, irrespective of the proportion of b-GP. The dynamics of the spin-labelled Insulin and its microviscosity inside the gels and during release were monitored by EPR spectroscopy. The results indicate that

														the Insulin was incorporated into the aqueous environment of the gel and was released in its native form. The in vitro drug release from the gels was governed by diffusion of drug from the gel matrix. A sustained release of Insulin was observed over a period of 2 weeks. Increasing the proportion of b-GP increased the amount of released Insulin and the velocity thereof

44	Preparation and characterization of insulin nanoparticles employing Chitosan and poly(methylmethacrylate/methylmethacrylic acid) copolymer	Li, Ming-Guang and Lu, Wan-Liang and Wang, Jian-Cheng and Zhang, Xuan and Zhang, Hua and Wang, Xue-Qing and Wu, Cui-Shuan and	2006	Ingenta Connect	China	Journal of Nanoscience and Nanotechnology	13	34	in vitro	Nanoparticles	Oral	Both insulin entrapment efficiency (62,04 to 72,57%) and loading percentage (0,87 to 3,10%) using chitosan-Eudragit L100-55 as matrix materials were the highest.	8 hours	As most of polypeptides are marginally stable, a mild formulation procedure would be beneficial for the activities of these drugs. The objective of the present study was to develop a novel pH-sensitive nanoparticle system that was suitable for entrapment of hydrophilic insulin but without affecting its conformation. Chitosan was incorporated as a positively charged material, and one of the three poly(methylmethacrylate/methylmethacrylic acid) copolymers, consisting of Eudragit L100-55, L100, and S100, was used as a negatively charged polymer for preparation of three insulin nanoparticles, respectively. Three nanoparticles obtained were spherical. The mean diameters were in the range from 200 nm to 250 nm, and the entrapment efficiencies, from 50% to 70%. The surface analysis indicated that insulin was evenly distributed in the nanoparticles. Polymer ratio of chitosan to Eudragit was the factor which influenced the nanoparticles significantly. Characterization results showed that the electrostatic interactions existed, thus providing a mild formulation procedure which did not affect

														the chemical integrity and the conformation of insulin. In vitro release studies revealed that all three types of the nanoparticles exhibited a pH-dependant characteristic. The modeling data indicated that the release kinetics of insulin was nonlinear, and during the release process, the nanoparticles showed a polynomial swelling. On overall estimation, the insulin chitosan-Eudragit L100-55 nanoparticles may be better for the Oral delivery. This new pH-sensitive nanoparticle formulation using chitosan and Eudragit L100-55 polymer may provide a useful approach for entrapment of hydrophilic polypeptides without affecting their conformation. Copyright © 2006 American Scientific Publishers. All rights reserved.

45	Preparation and characterization of novel derivatives of chitosan and trimethyl chitosan conjugated with dipeptides and vitamin B12 as candidates for Oral delivery of insulin	Omid, Nersi Jafary; Babanejad, Niloofar; Amini, Hossein; Amini, Mohsen; Rafiee Tehrani, Morteza; Dorkoosh, Farid	2014	Springer	Iran	Journal of Polymer Research	16	11	in vitro	Nanoparticles	Oral	The loading efficacy of the particles is low which may again be due to low solubility of the polymer.	6 hours	Chitosan and its derivatives are widely used in drug delivery systems due to their bio-degradebility, bio-compatibility and absorption enhancing properties. Many peptide and protein derived therapeutics cannot be administered through Oral rout because of the proteolytic condition of gastro-intestinal tract and their low bio-availability. Insulin is a peptide drug which is widely used in diabetics as repeated daily injection. Due to the fact that there are receptors for didpeptides and vitamine B12 in small intestine, in this research work novel derivatives of chitosan and trimethyl chitosan conjugated with glycyl-glycine, alanyl-alaninie and vitamine B12 were synthesized and characterized. The structure of conjugates as well as substitution of different functional groups was confirmed by different instrumental analytical methods such as Fourier transform infrared, magnetic resonance, and X-ray diffraction spectroscopy. Nano-particles of aforementioned loaded with insulin were prepared and their size, surface electrical charge and morphology characterized and their release profile were studied. The results

														are promising and reveal that these new chitosan and trimethyl chitosan derivatives are potential vehicles for protein and peptide drug molecules.

46	Optimization of pH-responsive carboxymethylated iota-carrageenan/chitosan nanoparticles for Oral insulin delivery using response surface methodology	Pratyusa Sahoo; Kok Hoong Leong ; Shaik Nyamathulla; Yoshinori Onuki; Kozo Takayama; Lip Yong Chung	2017	Elsevier	Malaysia/ Japan	Reactive and Functional Polymers	11	3	in vitro	Nanoparticles	Oral	The resulting optimized nanoparticles had loading capacity and entrapment efficiency of 10.7 ± 0.6%, and 86.9 ± 2.6%, respectively.	12 hours	In this study, we investigated the influence of hydrophobized chitosan on the formation and thermodynamic and surface tension properties of insulin–chitosan (I–Ch) polyelectrolyte complexes (PECs). We used an alkylation procedure to insert 12 carbon chains along the chitosan macromolecule with final substitution degrees of 5, 10, and 50%. NMR and IR spectroscopy were used to evaluate the success and extent of the hydrophobization procedure. Isothermal titration calorimetry (ITC) was used to determine the type and extent of the existing intermolecular interactions between the different constituting components of the insulin–hydrophobized chitosan PECs. Through the surface tension and diffusion coefficients at the air–water interface and ITC experiments with different I–Ch proportions, we demonstrated that around 34, 24, 25, and 60–80 insulin molecules saturated 0, 5, 10, and 50% hydrophobized chitosans, respectively. Surface tension experiments at the air–water interface demonstrated that the interaction of insulin molecules on the

														unmodified chitosan increased the hydrophobicity; this was mainly due to electrostatic interaction. On the contrary, insulin–hydrophobized chitosan interaction lowered the PEC hydrophobicity because of insulin alkyl chain interaction, and therefore, the hydrophilic insulin groups at the PEC surface contributed to a higher surface tension.
47	Probing insulin's secondary structure after entrapment into alginate/chitosan nanoparticles	B. Sarmento; D.C. Ferreira; L. Jorgensen; M. van de Weert	2007	Elsevier	Potugal/ Denmark	European Journal of Pharmaceutics and Biopharmaceutics	8	184	in vitro	Nanoparticles	Oral	Decreasing the alginate:chitosan mass ratio from 6:1 to 3.3:1 led to an increase in AE to 91% and a decrease of the LC to 6,5%.	2 hours	The aim of the present study was to probe the structural integrity of insulin after being entrapped into chitosan/alginate nanoparticles produced by ionotropic polyelectrolyte pre-gelation. By manipulating the alginate:chitosan mass ratio and the pH during nanoparticle production, desired nanoparticles with a mean size of 850 (±88) nm and insulin association efficiency of 81 (±2)% were obtained. Insulin secondary structure was assessed by Fourier transform

														infrared (FTIR) and circular dichroism (CD) after entrapment into nanoparticles and after release from the particles under gastrointestinal simulated conditions. FTIR second-derivative spectra and area-overlap compared to an insulin standard confirmed that no significant conformational changes of insulin occurred in terms of a-helix and b-sheet content. Far-UV-CD spectra corroborated the preservation of insulin structure during the nanoparticle production procedure. The presented nanoparticulate system is a promising carrier for insulin Oral delivery since it preserves insulin structure and therefore also, potentially, its bioactivity.

48	Oral delivery of insulin from alginate/chitosan crosslinked by glutaraldehyde	Djamel Tahtat; Mohamed Mahlous; Samah Benamer; Assia Nacer Khodja; Habiba Oussedik-Oumehdi; Fatima Laraba-Djebari	2013	Elsevier	Algeria	International Journal of Biological Macromolecules	9	65	in vitro	Beads	Oral	The entrapment of insulin increased with the increase of chitosan content in the beads. The loading efficiency were 1.86% for 8:2, 2,12% for 7:3 and 2,16% for 6:4.	6 hours	Insulin is mainly administered via subcutaneous route by injection which is the cause of painful and possible infections. Oral insulin administration would present a more convenient form of application because it is less invasive. Oral delivery of insulin to the gastrointestinal tract is one of the most challenging issues, because it numerous barriers to overcome in order to create an effective system for insulin delivery. In the present study, insulin-loaded alginate/chitosan blend gel beads were prepared with different mass ratios. Chitosan was depolymerized by gamma irradiation at a dose of 80 kGy reducing its molecular weight for ideal blend with sodium alginate. The homogeneous solution of alginate and chitosan was dripped into $CaCl_2$ solution (2%), the resultant calcium crosslinked beads were dipped in glutaraldehyde (2%) solution sequentially to prepare dual crosslinked beads with improved mechanical properties so as to withstand the simulated gastric fluid (SGF) and simulated intestinal fluid (SIF). Morphological structure, FTIR analysis, thermogravimetry analysis, specific

														surface area, gel fraction, swelling kinetics in SGF and SIF, loading efficiency, insulin release behavior, mucoadhesivity of the alginate/chitosan beads were investigated. The cumulative insulin release of pure alginate beads (10:0) reached as maximum level 100% in 3 h after they were dipped in SIF. Concerning the beads Alg/Chi (8:2), Alg/Chi (7:3) and Alg/Chi (6:4) the cumulative release of insulin reached 90.5%, 89.2% and 70.2%, respectively in 6 h. The rate of 100% was reached after 24 h for Alg/Chi (8:2), Alg/Chi (7:3) and after 73 h for Alg/Chi (6:4). The presence of chitosan in the blend beads decreased the cumulative insulin release in gastric media and enhanced behavior of alginate/chitosan beads in intestinal medium due to the crosslinking. The alginate/chitosan beads crosslinked by glutaraldehyde may be considered as potential insulin carriers for Oral drug delivery system.

49	Mechanism of surface charge triggered intestinal epithelial tight junction opening upon chitosan nanoparticles for insulin Oral delivery	Wang, Juan and Kong, Ming and Zhou, Zhenjin and Yan, Dong and Yu, Xiaoping and Cheng, Xiaojie and Feng, Chao and Liu, Ya and Chen, Xiguang	2017	Elsevier	China	Carbohydrate Polymers	7	19	In vivo	Nanoparticles	Oral	Not informed	13 hours	Intestinal epithelium is a major barrier limiting the absorption of Oral insulin owing to the presence of intercellular tight junctions (TJs). Previous studies proved that carboxymethyl chitosan/chitosannanoparticles (CMCS/CS-NPs) exhibited surface charge depending promotion of intestinal absorption. This study further confirmed the better performances of insulin:CMCS/CS-NPs(−) in enhancing epithelial permeation, increasing bioavailability and extending blood duration of insulin than insulin:CMCS/CSNPs(+). Immunohistochemistry sections found that TJs on jejunum epithelium completely disappeared in insulin:CMCS/CS-NPs(−) group, partially existed in insulin:CMCS/CS-NPs(+) group and appeared no change in control. Surface charges of CMCS/CS-NPs triggered intestinal epithelial TJs opening through different mechanisms. Although a down-regulation of TJs protein claudin-4 was detected in both nanoparticles groups, for phosphorylated claudin-4, the activating form, whose down-regulation occurred only in insulin:CMCS/CS-NPs(−) group. Counting

														upon synergetic effects of Ca2+ deprivation from adherens junctions and claudin-4 dephosphorylation and degradation, CMCS/CS-NPs(−) triggered more extensive disintegration of TJs and stronger paracellular permeability than the positive
50	Effective protection and controlled release of insulin by cationic - cyclodextrin polymers from alginate/chitosan nanoparticles	Nan Zhang; Jiahui Li; Wenfeng Jiang; Chunhong Ren; Jianshu Li; Jianyu Xin; Ke Li	2010	Elsevier	China	International Journal of Pharmaceutics	7	151	in vitro	Nanoparticles	Oral	The nanoparticles can load insulin with the association efficiency (AE) up to 87%.	6 hours	In an alginate/chitosan nanoparticle system, insulin was protected by forming complexes with cationic - cyclodextrin polymers (CPCDs), which were synthesized from - cyclodextrin (-CD), epichlorohydrin (EP) and choline chloride (CC) through a one-step polycondensation. Due to the electrostatic attraction between insulin and CPCDs, as well as the assistance of its polymeric chains, CPCDs could effectively protect insulin under simulated gastrointestinal conditions. The nanoparticles have their mean size lower than 350 nm and can load insulin with the association efficiency

														(AE) up to 87%. It is notable that the cumulative insulin release in simulated intestinal fluid was significantly higher (40%) than that without CPCDs (18%) because insulin was mainly retained in the core of the nanoparticles and well protected against degradation in simulated gastric fluid. Far-UV circular dichroism analysis also corroborated the preservation of insulin structure during the nanoparticle preparation and release process.
51	Preparation and evaluation of chitosan-ethylenediaminetetra acetic acid hydrogel films for the mucoadhesive transbuccal delivery of insulin	Fuying Cui,Chunbai He, Miao He,Cui Tang, Lichen Yin,Feng Qian,Chunhua Yin.	2008	Wiley Online Library	China	Journal of Biomedical Materials Research Part A	8	46	In vivo	Films	Transbuccal	Insulin loaded films with the dose of 5,14 and 83 IU/Kg	5 hours	This manuscript describes the development of a new porous, flexible bilaminated film for buccal protein administration by a simple and mild casting procedure. It consists of a mucoadhesive layer (chitosan-ethylenediaminetetraacetic acid hydrogel film) containing protein drugs and an impermeable protective layer made of ethylcellose. The obtained mucoadhesive layer was characterized in terms of Fourier transform infrared spectroscopy, rheology, swelling, and mucoadhesion. Rheology results showed that chitosan-ethylenediaminetetraacetic acid hydrogel (10:2) possessed the greatest degree of

														viscoelasticity and was well-structured compared with other hydrogels. The in vitro mucoadhesion studies also showed that the mucoadhesive force of the hydrogel remained over 17,000 N/m2 during 4 h in the simulated oral cavity. The insulin loaded bilaminated film showed a pronounced hypoglycemic effect following buccal administration to healthy rats, achieving a 17% pharmacological availability compared with subcutaneous insulin injection. According to these results, the bilaminated film would be a promising delivery carrier for protein drugs via the buccal route

52	Dual Stimuli-Responsive Nanoparticle-Incorporated Hydrogels as an Oral Insulin Carrier for IntestineTargeted Delivery and Enhanced Paracellular Permeation	Liang Liu, Ying Zhang, Shuangjiang Yu, Zhiming Yang, Chaoliang He, and Xuesi Chen	2018	ACS Publication	China	ACS Biomaterials Science & Engineering	44	0	In vivo	Nanoparticle	Oral	Encapsulating insulin into chitosan/insulin/heparin nanoparticles 30 UI/Kg	12 hours and 21 days	For enhanced Oral insulin delivery, a strategy of acid-resistant and enteric hydrogels encapsulating insulin-loaded nanoparticles was developed. The nanoparticles were prepared by the formation of an anionic insulin/heparin sodium (Ins/HS) aggregate, followed by coating of chitosan (CS) on the surface. The nanoparticles, tagged as CS/Ins/HS NPs, exhibited excellent mucosa affinity, effective protease inhibition and marked paracellular permeation enhancement. Moreover, to improve the acid-stability of CS/Ins/HS NPs and impart the capacity of intestine-targeted delivery, a pH- and amylase-responsive hydrogel was synthesized via free radical copolymerization, using methacrylic acid as the monomer and acrylate-grafted-carboxymethyl starch as the crosslinker. The resulting hydrogel exhibited sharp pH-sensitivity in gastrointestinal tract and rapid enteric behavior under intestinal amylase. The additional protection for insulin in artificial gastric fluid was confirmed by packaging CS/Ins/HS NPs into the hydrogel. The obtained nanoparticle-

														incorporated hydrogel was named as NPs@Gel-2. The release of insulin from NPs@Gel-2 was evidently accelerated in artificial intestinal fluid containing α-amylase. Furthermore, the hypoglycemic effects were evaluated with type-1 diabetic rats. Compared to subcutaneous injection of insulin solution, the relative pharmacological availability (rPA) for Oral intake of NPs@Gel-2 (30 IU/kg) was determined to be 8.6%, along with rPA of 4.6% for Oral administration of unpackaged CS/Ins/HS NPs (30 IU/kg). Finally, the two-week therapeutic outcomes in diabetic rats were displayed after twice-daily treatments by Oral intake of NPs@Gel-2, showing the relief of diabetic symptoms and suppression of weight loss in the rats. Therefore, this dual stimuli-responsive nanoparticle-incorporated hydrogel system could be a promising platform for Oral insulin delivery.

53	Low Molecular Weight Chitosan-Insulin Complexes Solubilized in a Mixture of Self-Assembled Labrosol and Plurol Oleaque and Their Glucose Reduction Activity in Rats	Amani M. Elsayed, Aseel H. Khaled, Mayyas M. Al Remawi, Nidal A. Qinna, Hussam Abu Farsakh and Adnan A. Badwan,	2018	MDPI	Saudi Arabia/Jordan	Marine Drug	15	2	In vivo	Nanoparticles	Oral	Not informed	12 hours	Oral insulin delivery that better mimics physiological pathways is a necessity as it ensures patient comfort and compliance. A system which is based on a vehicle of nano order where positively charged chitosan interacts with negatively charged insulin and forms a polyelectrolyte complex (PEC) solubilizate, which is then solubilized into an oily phase of oleic acid, labrasol, and plurol oleaque-protects insulin against enzymatic gastrointestinal reduction. The use of an anionic fatty acid in the oily phase, such as oleic acid, is thought to allow an interaction with cationic chitosan, hence reducing particle size. Formulations were assessed based on their hypoglycaemic capacities in diabetic rats as compared to conventional subcutaneous dosage forms. 50 IU/kg Oral insulin strength could only induce blood glucose reduction equivalent to that of 5 IU/kg (1 International unit = 0.0347 mg of human insulin). Parameters that influence the pharmacological availability were evaluated. A preliminary investigation of the mechanism of absorption suggests

														the involvement of the lymphatic route.
54	Biodistribution, pharmacodynamics and pharmacokinetics of insulin analogues in a rat model: Oral delivery using pH-Responsive nanoparticles vs. subcutaneous injection	Kiran Sonaje, Kun-Ju Lin, Shiaw-Pyng Wey, Che-Kuan Lin, Tzyy-Harn Yeh, Ho-Ngoc Nguyen, Chia-Wei Hsu, Tzu-Chen Yen, Jyuhn-Huarng Juang, Hsing-Wen Sung,	2010	Elsevier	Taiwan	Biomaterials	9	158	In vivo	Nanoparticles	Oral	The formulations were released at pH 2.5, the cumulative amount of aspart-insulin released from test NPs was about 20%, while it was approximately 35% at pH 6.6.	24 hours	In this study, we report the biodistribution of aspart-insulin, a rapid-acting insulin analogue, following Oral or subcutaneous (SC) administration to rats using the single-photon emission computed tomography (SPECT)/computed tomography (CT). Oral delivery of aspart-insulin was achieved using a pH-responsive nanoparticle (NP) system composed of chitosan (CS) and poly(g-glutamic acid). The results obtained in the SPECT/CT study indicate that the Orally administered aspart-insulin was absorbed into the systemic circulation, while the drug carrier (CS) was mainly retained in the gastrointestinal tract.Via the SC route, the peak aspart-insulin concentration in the peripheral tissue/plasma was observed at 20 min

														after injection. Within 3 h, half of the initial dose (ID) of aspart-insulin was degraded and excreted into the urinary bladder. In contrast, via Oral delivery, there was constantly circulating aspart-insulin in the peripheral tissue/plasma during the course of the study, while 20% of the ID of aspart-insulin was metabolized and excreted into the urinary bladder. In the pharmacodynamic (PD) and pharmacokinetic (PK) evaluation in a diabetic rat model, the Orally administered aspart-insulin loaded NPs produced a slower hypoglycemic response for a prolonged period of time, whereas the SC injection of aspartinsulin produced a more pronounced hypoglycemic effect for a relatively shorter duration. Finally, comparison of the PD/PK profiles of the Orally administered aspart-insulin with those of the SC injection of NPH-insulin, an intermediate-acting insulin preparation, suggests the suitability of our NP system to be used as a non-invasive alternative for the basal insulin therapy.

55	Encapsulation of insulin in chitosan-coated alginate beads: Oral therapeutic peptide delivery	Seçil Önal; Figen Zihnioglu	2002	Taylor & Francis	Turkey	Journal Artificial Cells, Blood Substitutes, and Biotechnology	9	50	in vitro	Beads	Oral	not informed	6 hours	Insulin was encapsulated in calcium alginate beads coated with chitosan. Its release from alginate-chitosan and alginate-chitosanglutaraldehyde beads was studied in artificial gastric (pH 1.2) and intestinal (pH 7.5) fluids. By comparing the release amounts, the ionic interaction between alginate-chitosan matrix with the medium pH's, intestinal fluid was found to be the better. The degradation of released insulin was also searched, even after 6 h incubation, the beads remained stable and the undegraded insulin seemed to be sufficient for the physiological conditions. Consequently, it can be said that the system can be offered for Oral delivery of the therapeutic peptide drug insulin.

Strings of search*:

PubMed: ((((chitosan) AND insulin) AND ((delivery system) OR controlled delivery system))

Science Direct: ((((chitosan) AND insulin) AND ((delivery system) OR controlled delivery system))

Engineering Village: (1) chitosan and insulin and controlled delivery system

(2) (chitosan and insulin and delivery system)

HubMed: ((((chitosan) AND insulin) AND ((delivery system) OR controlled delivery system))

***** All the searches were performed in the search advanced model of the websites.

Start tool:

Start tool (StArt 2.3,4.2) is available to download in http://lapes.dc.ufscar.br/tools/start_tool .
